# Oridonin from *Rabdosia rubescens*: An emerging potential in cancer therapy – A comprehensive review

**DOI:** 10.1002/fsn3.3986

**Published:** 2024-02-01

**Authors:** Muhammad Asif Ali, Noohela Khan, Ahmad Ali, Hira Akram, Noushaba Zafar, Kinza Imran, Tooba Khan, Khushbukhat Khan, Muhammad Armaghan, Marta Palma‐Morales, Celia Rodríguez‐Pérez, Angela Caunii, Monica Butnariu, Solomon Habtemariam, Javad Sharifi‐Rad

**Affiliations:** ^1^ Department of Food Science and Human Nutrition UVAS Lahore Pakistan; ^2^ Department of Nutrition Sciences Rashid Latif Medical College Lahore Pakistan; ^3^ Department of Healthcare Biotechnology, Atta‐ur‐Rahman School of Applied Biosciences National University of Sciences and Technology Islamabad Pakistan; ^4^ Cancer Clinical Research Unit, Trials360 Lahore Pakistan; ^5^ Departamento de Nutrición y Bromatología, Facultad de Farmacia Universidad de Granada Granada Spain; ^6^ Instituto de Nutrición y Tecnología de los Alimentos ‘José Mataix’ Universidad de Granada Granada Spain; ^7^ Instituto de Investigación Biosanitaria de Granada (ibs.GRANADA) Granada Spain; ^8^ “Victor Babes” University of Medicine and Pharmacy Timisoara Romania; ^9^ University of Life Sciences "King Mihai I" from Timisoara Timisoara Romania; ^10^ Pharmacognosy Research & Herbal Analysis Services UK University of Greenwich Kent UK; ^11^ Facultad de Medicina Universidad del Azuay Cuenca Ecuador

**Keywords:** anticancer properties, cancer treatment, diterpenoid, natural compound, ORI

## Abstract

Cancer incidences are rising each year. In 2020, approximately 20 million new cancer cases and 10 million cancer‐related deaths were recorded. The World Health Organization (WHO) predicts that by 2024 the incidence of cancer will increase to 30.2 million individuals annually. Considering the invasive characteristics of its diagnostic procedures and therapeutic methods side effects, scientists are searching for different solutions, including using plant‐derived bioactive compounds, that could reduce the probability of cancer occurrence and make its treatment more comfortable. In this regard, oridonin (ORI), an ent‐kaurane diterpenoid, naturally found in the leaves of *Rabdosia rubescens* species, has been found to have antitumor, antiangiogenesis, antiasthmatic, antiinflammatory, and apoptosis induction properties. Extensive research has been performed on ORI to find various mechanisms involved in its anticancer activities. This review article provides an overview of ORI's effectiveness on murine and human cancer populations from 1976 to 2022 and provides insight into the future application of ORI in different cancer therapies.

## Graphical abstract

Oridonin (ORI), an ent‐kaurane diterpenoid, naturally found in the leaves of *Rabdosia rubescens* species, has been found to have antitumor and antiangiogenic properties. It inhibits several cancer‐signaling pathways like NFκB, PI3K, BCL2, BCLX, and p53‐mediated pathways. It showed anticancer activity in different cancer cell lines and more research needs to be done for improved bioavailability of ORI.

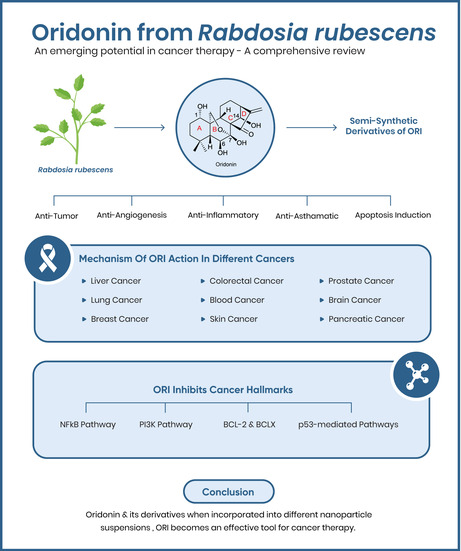

## 1 INTRODUCTION

Cancer is one of the major public health problems in the world. In 2019, 23.6 million newly identified cancer cases, 10.0 million cancer‐related deaths, and cancer‐attributable disability‐adjusted life years (DALYs) of approximately 250 million were recorded worldwide. This reflects an increase in new cases by 26.3%, a death rate by 20.9%, and DALYs by 16.0% since 2010 (Kocarnik et al., 2022).

According to the WHO, the most prevailing types of tumors in the year 2020 were breast cancer (a total of 2.26 million cases), lung cancer (2.21 million cases), colon and rectal cancer (1.93 million newly diagnosed cases), prostate cancer (1.41 million cases), nonmelanoma skin malignancy (a total of 1.20 million affected persons), and gastric carcinoma (1.09 million incidences) (Sung et al., 2021). Lung cancer accounted for 1.80 million fatalities in 2020, followed by colon and rectal tumors (916,000 deaths), malignancy of the liver (in total 830,000 demises), gastric cancer (769,000 fatalities), and breast carcinoma (685,000 demises). Similarly, the survey by GLOBOCON indicated that approximately 10 million deaths occurred due to cancer in 2020 (Sung et al., 2021).

Each cancer type requires its own treatment plan that can only be implemented after an accurate diagnosis. Usually, systemic therapies such as chemotherapy or hormonal therapy, radiotherapy, targeted biological therapy, and surgery are used in cancer treatment. To achieve a favorable therapeutic outcome, it is critical to complete the treatment regimen within the allocated time period (Piñeros et al., 2021). However, rising rate of cancer drug resistance has jeopardized the chemotherapeutic treatment efficacy. Therefore, efforts are being made in identifying unique molecular targets for improving diagnosis (Khan et al., 2022; Khan, Safi, et al., 2020; Shabbir et al., 2022), using natural compounds as chemopreventive agents, or novel therapeutic compounds for cancer management (Butt et al., 2021; Khan, Quispe, et al., 2020; Shabbir et al., 2021). In this regard, an ent‐kaurane diterpenoid, ORI, first isolated from the leaves of *Rabdosia rubescens* may serve as a lead candidate.

ORI modulates the progression of cell cycle and induces autophagy and phagocytosis, which ultimately results in apoptosis. Through this mechanism, it exerts remarkable anticancer action in a variety of cancer types (Liu et al., 2021). Due to its unique molecular structure, ORI has been shown to exhibit antiproliferative effect in over 20 human cancer cell lines, such as cancers of the esophagus (Song et al., 2019), lung (Li, Wang, Shen et al., 2018), liver (Zhang et al., 2006), prostate (Ming et al., 2016), breast (Zhang et al., 2006), and colon and rectal cancer (Zhang et al., 2019). Furthermore, ORI has been found to decrease cancerous cell growth and proliferation by inducing apoptosis while also inhibiting metastasis in human breast and ovarian cancer cells (Wang & Zhu, 2019).

Considering its unique ability to curb cancer, the aim of this review was to summarize the potential use of ORI in cancer and to describe the molecular pathway targeted by it. The review demonstrated the clinical importance of ORI and identified the research gaps that need to be filled to increase its applicability at clinical level.

## 2 DESCRIPTION OF THE PLANT SOURCE

### 2.1 Plant source

ORI is the main diterpenoid isolated from the plant *R. rubescens* which is also known by a synonym *Isodon rubescens* and *R. dichromophylla* (List, 2013). The genus includes more than 150 perennial herbs species. The plant is also locally known as “Binglingcao,” “Donglingcao,” and “Shanxiangcao” in China (Chen et al., 2022).

### 2.2 Geographical distribution

*Rabdosia rubescens* is distributed extensively in the Yellow River and Yangtze River basins located in China. Specifically, the focal area of its production is situated in the south of Taihang Mountain in Jiyuan, He'nan, and had 1400 hectares cultivation in the year of 2015. It is recognized as “National Geographical Indication Protected Product” since 2006 (Chen et al., 2022).

### 2.3 Phytochemical constituents

*Rabdosia rubescens* present several secondary metabolites which include di‐ and triterpenoids, phenolic compounds, alkaloids, essential oils, and other compounds. Among these, diterpenoids are the main components with the most biologically active compounds represented by ORI. These compounds display anti‐inflammatory, antioxidant, antitumor, antibacterial, antidementia, anticardiovascular, and immunomodulatory activities (Chen et al., 2022).

### 2.4 General molecular features of ORI

Diterpenoids have become one of the most important groups of natural products due to their distinct biological properties and pharmaceutical application. In terms of cancer, their best representative is Paclitaxel (Taxol) which along with other taxane‐type diterpenoids have been successfully applied in clinical settings (Malik et al., 2018). As a kaurane‐type diterpenoid, ORI has recently gained a lot of interest for its outstanding pharmacological properties and its safety to be used in complementary therapies. The principal natural source of ORI, *R. rubescens*, has also been used in traditional Chinese medicine to treat inflammation and cancer in many Asian countries (Tan et al., 2011).

**FIGURE 1:**
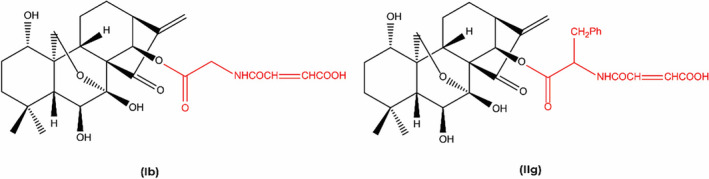
Structural isomers of oridonin.

The structural attributes for the biological activity of ORI have been studied. Most importantly, removing the characteristic molecular ring or methylene saturation can compromise the anticancer effect of ORI, which is dependent on the methylene cyclopentanone (enone) on the D‐ring (Figure 1) (Ding et al., 2013a). Additionally, C (17) is electrophilic due to the hydrogen bonding between 6‐hydroxy and 15‐carbonyl group, which enhances its attraction to electrophilic enzymes in tumor cells. The 7‐hydroxyl group intensifies the nucleophilic property of the parallel 14‐hydroxyl group, whose esterification might increase the anticancer action. Removal of the 7‐hydroxyl group decreases the antitumor activity of ORI (Node et al., 1983).

ORI, however, has low bioavailability and poor water solubility that may limit its therapeutic potential. So, studies are attempting to improve its pharmacokinetics properties by modulating its structure (Liu et al., 2011). One study demonstrated the process for synthesizing a stable ORI nanosuspension (ORI‐N) with improved drug saturation solubility and dissolving rate, using the high‐pressure homogenization technique (Gao et al., 2007). The outcomes indicating an improvement in ORI solubility and bioavailability are promising (Gao et al., 2007).

## 3 CURRENT MEDICAL APPLICATIONS – OFFICIAL TREATMENT OR TRADITIONAL MEDICINE

Recent studies show that ORI has many great pharmacological properties such as anticancer (Hu et al., 2020; Jeon et al., 2019; Vasaturo et al., 2018), anti‐inflammatory (He et al., 2018), as well as protective activities against hepatorenal and cardioprotective exercises (Li et al., 2021).

ORI might serve as an antitumor agent in several types of cancer (breast cancer [Li et al., 2019], HCC [Li, Mu, et al., 2020], gall bladder cancer [Chen et al., 2019], cervical cancer [Hu et al., 2007], leukemia [Li & Ma, 2019; Zhang et al., 2019], etc.) as it is found capable of inhibiting cell division and activating apoptosis cascade in cancerous cells (Li et al., 2021). It induces autophagy in cancerous colorectal cells (CRC) by inhibiting glucose metabolism (Yao et al., 2017). ORI has also been observed to promote apoptosis of uveal melanoma cells by downregulating FAS protein (Gu et al., 2015). Also, ORI has anti‐inflammatory and cytoprotective role that has been demonstrated using in vitro and in vivo models (Du et al., 2008; Wang, Yang, et al., 2014; Zhang, Daniels, & Schluesener, 2013). One study showed that ORI has both protective and therapeutic role in mouse models of type 2 diabetes, gouty joint inflammation, and peritonitis as it downregulates the activation of nucleotide‐binding domain, leucine‐rich–containing family, pyrin domain–containing‐3 (NLRP3) (He et al., 2018). ORI also has the potential to downregulate the activation of glial cells and inhibit the excretion of cytokines (nitric oxide [NO], interleukin‐1 beta [IL‐1β], IL‐6, and tumor necrosis factor‐alpha [TNF‐α]) (Wang, Yang, et al., 2014). Furthermore, ORI enhanced nerve nuclear growth factor expression which ultimately promotes survival and differentiation of neurons in mice with Alzheimer's disease. This study highlighted the protective and antineuroinflammatory influence of ORI in neurological diseases (Wang, Yang, et al., 2014).

ORI is also a potent liver protective compound, however its impact on liver steatosis is still unknown. Therefore, to study the effect of free fatty acids on liver, a basic model of liver steatosis was developed by Cheng et al. (2021). Total lipid content, especially triglycerides, was estimated after the addition of ORI to the free fatty acid cell model for 24 h. Furthermore, studies revealed that ORI can enhance autophagy and decrease the liver cells steatosis (Cheng et al., 2021). Similarly, ORI, combined with ginsenoside and the fungus *Ganoderma lucidum*, is reported to enhance immunological processes and ultimately the apoptosis in hepatocellular carcinoma (HCC) cells (He et al., 2021).

Studies provide insight into underlying mechanism of kidney‐defensive job of ORI in acute kidney injury (AKI) and the basic system by which ORI further develops AKI in vivo and restrains irritation in lipopolysaccharide‐prompted bone marrow‐determined macrophages in vitro. ORI treatment enabled the development of serum creatinine and blood urea nitrogen levels in a mouse model of AKI. In addition, ORI enhances AKI wounds by stifling protein kinase B (AKT)‐intervened stimulating reaction of macrophages (Yan et al., 2020).

The mixture of salvianolic acid A, ORI, and epigallocatechin gallate is found to be effective against coronavirus as it directly targets the 3C‐like protease (3CLpro) and thus inhibits replication and transcription of the virus. Hence, ORI has demonstrated to be highly effective as antiviral in therapies for treating coronavirus diseases (Zhong et al., 2022).

Despite ORI has demonstrated to have numerous therapeutic properties, no side effects, or resistance development, further research for a better understanding about its mechanism of action involved in the protection against chronic illnesses is still needed (Liu et al., 2021).

An extensive review of literature was done through different search engines including PubMed and Google Scholar from the year 1976 to 2022. In total, 350 articles were reviewed for this purpose and further186 studies were added to explain the effect of ORI as an anticancer compound on different types of cancers such as lung, liver, breast, and prostate cancer. This literature review includes both in vitro and in vivo analysis conducted to demonstrate ORI's therapeutic impact on different cancers.

## 4 SEMISYNTHETIC DERIVATIVES OF ORI

**FIGURE 2:**
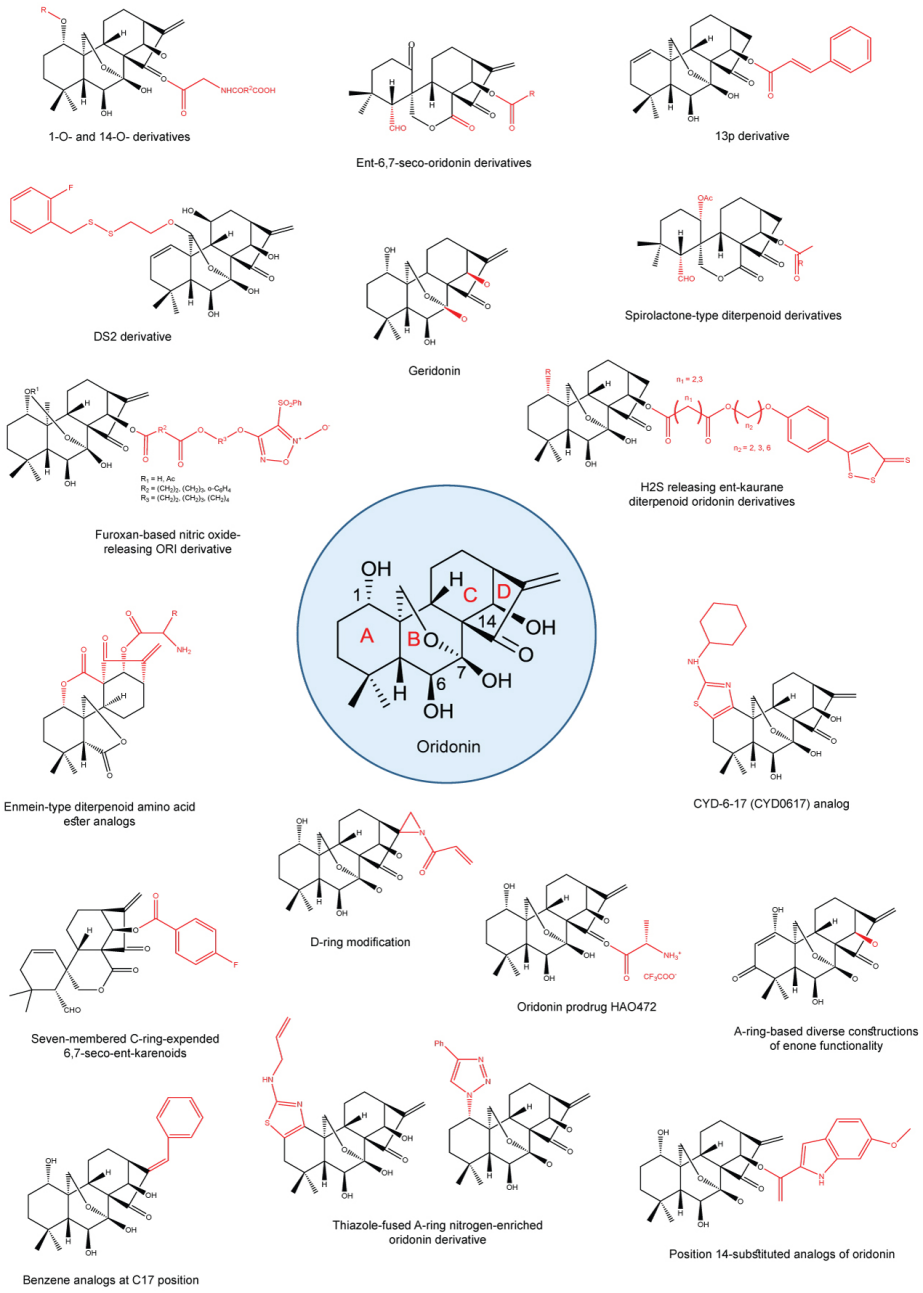
Different derivates of ORI. The main structure of ORI is enclosed in a blue circle and its derivatives surround it where functional groups are shown in red. Chemical structures are drawn using ChemDraw to show how different functional groups can change the role of primary compound and improve their solubility and bioavailability.

Different ORI derivatives have been found to have noticeable antitumor activity against different cancers. The structures of ORI derivatives are depicted in Figure 2 and their anticancer effect is displayed in Table 1.

**TABLE 1:** ORI derivatives along with their anticancer properties.

Sr. No.	Derivative	Cancer type	Main mechanism of action	IC_50_ values	References
(a)	1‐O‐ and 14‐O‐derivatives	Stomach cancer, leukemia, colon cancer, melanoma, liver cancer, lung cancer	Antiproliferative activity	0.84 μM for HL‐60 cell and 1.00 μM for BEL‐7402 cell	Xu et al. (2008)
(b)	Ent‐6,7‐seco‐ORI derivatives	Lung cancer, liver cancer, stomach cancer	Antiproliferative activity	0.55 μM	Xu, Yao, Hu, et al. (2017)
(c)	DS2 derivative	Esophagus cancer	Mitochondria‐mediated apoptosis	4.12 μM for MGC‐803	Ma et al. (2016)
(d)	13p derivative	Breast cancer	Apoptosis	0.220–1.997 μM	Xu, Yao, Luo, et al. (2017)
(e)	Furoxan‐based NO‐releasing ORI derivative	Myelogenous leukemia, stomach cancer, liver cancer	Antiproliferative activity	1.82 μM for K562, 1.81 μM for MGC‐803, and 0.86 μM for Bel‐7402	Shen et al. (2018)
(f)	Geridonin	Stomach cancer	Apoptosis, elevated reactive oxygen species (ROS) levels	10.40 for MGC‐803	Wang et al. (2017)
(g)	Enmein‐type diterpenoid amino acid ester analogs	Myeloid leukemia, stomach cancer, liver cancer	Antiproliferative activity	0.85–1.97 mM	Hu et al. (2019)
(h)	Spirolactone‐type diterpenoid derivatives	Myeloid leukemia, stomach cancer, liver cancer	Antiproliferative activity	0.39 mM and 1.39 mM	Li, Han, Tian, et al. (2016)
(i)	H_2_S releasing ent‐kaurane diterpenoid ORI derivatives	Colon cancer, liver cancer	Antiproliferative activity	2.57 μM for HepG2	Li, Chen, et al. (2020)
(j)	Seven‐membered C‐ring‐expended 6,7‐seco‐ent‐karenoids	Breast cancer	Apoptosis, cell cycle arrest	0.55 μM	Xu, Yao, Hu, et al. (2017)
(k)	Thiazole‐fused A‐ring nitrogen‐enriched ORI derivative	Breast cancer	Antiproliferative effect	0.1–1 μmol/L	Ding et al. (2013a)
(l)	CYD‐6‐17 (CYD0617) analog	Kidney cancer	Antichemoresistance effect, apoptosis	0.43 ± 0.05 μM	Zhou et al. (2017)
(m)	Analogs with D‐ring modification	Breast cancer	Apoptosis	10 μM	Li, Wang, Shen, et al. (2018) , Li, Wang, Ding, et al. (2018)
(n)	A‐ring‐based diverse constructions of enone functionality	Breast cancer	Apoptosis	28.0 ± 1.40 μM for MDA‐MB‐231	Ding et al. (2013a)
(o)	ORI prodrug HAO472	Lung cancer	Antiproliferative activity	5.0 mg/kg or 7.5 mg/kg in mice	Liu et al. (2016)
(p)	Benzene analogs at C17 position	Stomach cancer, liver cancer	Antiproliferative effect	1.05 μM for HCT116	Shen et al. (2018)
(q)	Position 14‐substituted analogs of ORI	Breast cancer, colon cancer, liver cancer	Antiproliferative effect	0.16 μM for HCT116	Shen et al. (2019)

In an in vitro trial, the ORI 1‐*O*‐ and 14‐*O*‐derivatives were synthesized and evaluated. The results of the study demonstrated that ORI derivatives showed cytotoxicity in various cancer cell lines including BGC‐7901 (stomach cancer), HL‐60 (promyelocytic leukemia), SW‐480 (colon cancer), B16 (melanoma), BEL‐7402 (human liver cancer), and A549 (lung cancer) (Xu et al., 2008). The study also disclosed that all ORI derivatives tested were efficient against the abovementioned cancer cell lines in vitro. However, two structural isomers of ORI, that is, ORI Ib and IIg were reported to be more effective with higher antitumor activity than ORI (the parent compound) and 5‐fluorouracil (positive drug) as IC_50_ value of 0.84 μM and 1.00 μM was observed for Ib and IIg in HL‐60 and BEL‐7402 cells, respectively (Xu et al., 2008).

Ent‐6,7‐seco‐ORI derivatives have been found to markedly exhibit antiproliferative activity against cancer cells such as A549, Bel‐7402, and MGC‐803 with IC_50_ value of 0.55 μM (Wang et al., 2012). The anticancer activity of the ORI derivative, DS2, has also been reported. Concretely, DS2‐treated cancer cells (IC_50_ value of 4.12 μM for MGC‐803) have shown to undergo mitochondria‐mediated apoptosis related to reactive oxygen species (ROS) generation and B‐cell lymphoma 2 (BCL‐2) associated X (BAX) regulation. The effect was observed in human esophageal cancer cell lines EC9703 and EC109 (Ma et al., 2016). The furaxon‐based NO‐donor hybrid compounds exhibited in vitro potential antitumor activity in cancerous cell lines including K562 (IC_50_ 1.82 μM), MGC‐803 (IC_50_ 1.81 μM), and Bel‐7402 (IC_50_ 0.86 μM) through producing high NO levels (Li, Wang, Ye, & Li, 2012). On their behalf, its benzene analogs at C17 position showed downregulation of cancerous cell proliferation in MGC803, A549, Bel7402, HCT116, AGS, and HeLa cell linings with IC_50_ value of 1.05 μM (Shen et al., 2018). Geridonin plays an effective role in the inhibition of stomach cancer cells both in vitro and in vivo. In this regard, one study showed that geridonin triggered apoptosis and elevated the intracellular ROS levels (Wang et al., 2017). Geridonin treatment combined with paclitaxel inhibited gastric tumor cells proliferation by upregulating the ROS‐mediated pathways (Li, Han, Tian, et al., 2016). Similarly, enmein‐type diterpenoid amino acid ester analogs showed antiproliferative action with dosage of 0.85–1.97 mM against Bel‐7402, PC‐3, SGC‐7901, K562, HL‐60, and A549 tumor cell lines through mitochondria‐related caspase‐dependent pathway (Hu et al., 2019).

Spirolactone‐type diterpenoid derivatives of ORI can release NO and were shown to have more potency and stronger antiproliferative activity in human tumor Bel‐7402, MGC‐803, K562, and CaEs‐17 cell lines through the alteration of mitochondria‐related pathways (Li, Han, Tian, et al., 2016). ORI derivatives, such as spirolactone‐type diterpenoids, exhibited enhanced apoptosis and antiproliferative activities in human hepatoma Bel‐7402 cells. Compared with Taxol, composite 12j disclosed more potency against Bel‐7402 and K562 cells with IC_50_ values of 1.39 μM and 0.39 μM, subsequently (Dal Piaz et al., 2013). Likewise, H_2_S releasing ent‐kaurane diterpenoid ORI derivatives are more potent than ORI in inducing antiproliferative activity in HepG2, HCT‐116, and K562 cancerous cells through the modification of both extrinsic and intrinsic apoptotic pathways (Li, Mu, et al., 2020). Position 14‐substituted analogs were found to have antiproliferative effects against HCT116, MCF7, and Bel7402 by inducing apoptosis through the regulation of p53‐MDM2 signaling pathway (Shen et al., 2019). HAO472 is an ORI prodrug that contains amino acid residue. A study based on observation of the effect of HAO472 against the inflammatory bowel disease disclosed that the prodrug is highly effective in treating colitis. HAO472 successfully suppressed the progression in trinitrobenzene sulfonic acid‐induced colitis mouse model by suppressing activated T cells, inflammation, and nuclear factor kappa B (NF‐κβ) pathway (Liu et al., 2016). CYD‐6‐17 analogs have the potential to inhibit the growth of different drug‐resistant renal cell carcinoma cells through PDPK1/Akt pathway regulation (Zhou et al., 2017).

Some derivatives of ORI have also demonstrated effective anticancer activity in breast cancer cells. Its 13p derivative was found to be 200 folds more potent than ORI in inducing apoptosis and thus cell cycle arrest in breast cancer cell lines (MCF‐7) at G2/M phase. Post 13‐p treatment, a decrease in membrane potential of mitochondria and an increase in BAX/BCL‐2 ratio, accompanied by activated caspase‐3 cleavage suggested that the mitochondrial pathway was involved in the 13p‐mediated apoptosis (Xu, Yao, Luo, et al., 2017). Other ORI novel derivatives, that is, seven‐membered C‐ring‐expended 6,7‐seco‐ent‐karenoids can hinder the proliferation of tumor cells by triggering apoptosis and cell cycle arrest in human breast cancer MCF‐7 cells (Xu, Yao, Luo, et al., 2017). Similarly, another novel derivate, A‐ring‐based diverse constructions of enone, has shown to significantly induce apoptosis and impede colony formation in cells of breast cancer (Ding et al., 2013a). Moreover, thiazole‐fused A‐ring nitrogen‐enriched ORI derivative has proved a strong antiproliferative action against MDA‐MB‐231 breast cancer cells (Ding et al., 2013a). Finally, the in vitro and in vivo analyses on breast cancer cell line disclosed that ORI D‐ring aziridinated analog can downregulate the growth of tumor cells through the increase in apoptosis and by inhibiting the formation of colony (Ding et al., 2018). Jesridonin (a product obtained from structural modification of ORI) has been found to show extensive anticancerous activity in both in vitro and in vivo xenograft mice model of EC109 cells via death receptor and mitochondrial pathways by downregulating E3 ubiquitin protein ligase MDM2, p53 gene, and BCL‐2 family members, and Caspase‐3/‐8/‐9 activation (Wang et al., 2015).

## 5 ORI IMPACT ON SEVERAL HALLMARKS OF CANCER

**FIGURE 3:**
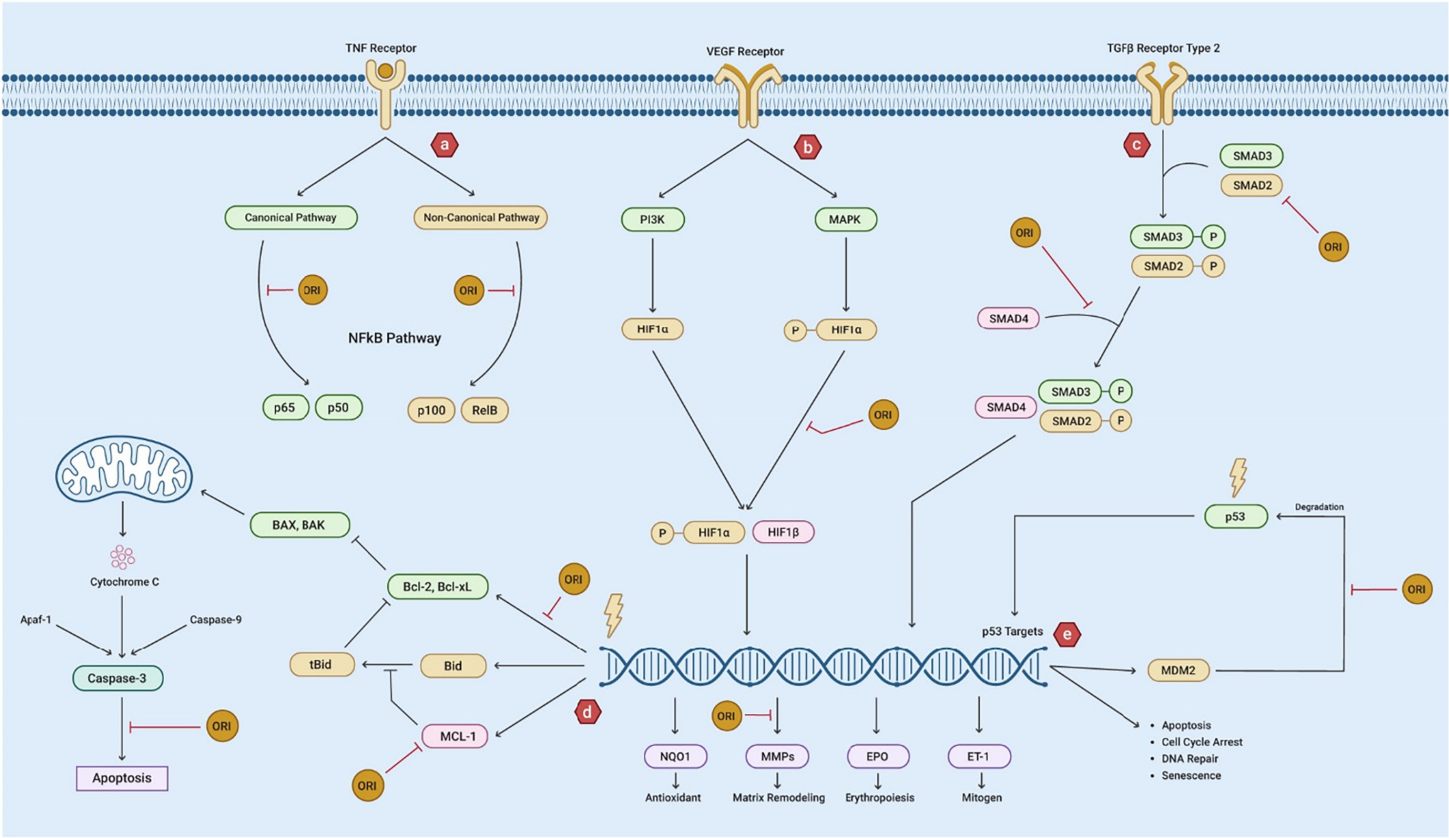
Molecular mechanism of anticancer effect of ORI. (a) ORI inhibits binding of NFκB transcription factors to its target genes. (b) VEGF receptors induce PI3K and MAPK signaling pathways to cause phosphorylation of HIF‐1α. ORI inhibits the entry of HIF‐1α into the nucleus. It also inhibits the transcription of MMPs that normally cause matrix remodeling. (c) Signaling through TGFβ type 2 receptors phosphorylate SMAD2 and SMAD3. ORI inhibits phosphorylation of SMAD2 and binding of SMAD4 with the complex that enters the nucleus to regulate invasion, inflammation, and proliferation. (d) ORI inhibits the transcription of antiapoptotic proteins BCL2 and BCLX. It prevents MCL‐1 to convert BID into tBID which inhibits the function of BCL2 proteins. ORI also inhibits caspase‐3 to induce apoptosis. (e) p53 target genes transcript into MDM2 that can again bind with p53 to cause its degradation. ORI inhibits the binding of MDM2 with p53.

ORI anticancer activity has been reported by several studies. It targets numerous cancer hallmarks to promote the cancer cell death and invasiveness inhibition. Figure 3 contains the information regarding the molecular mechanism of action of ORI in cancer.

### 5.1 Cell cycle arrest and cancer cell death induction

ORI induce the signal transduction from cellular pathways that are involved in promoting cell death in cancer. It initiates a mitochondria‐dependent and mitogen‐activated protein kinase (MAPK)‐dependent cell death and the mechanism is caspase independent (Zhang et al., 2004b). ORI exhibits dose‐ and time‐dependent inhibition of the cancerous cell growth and upregulation of apoptosis in blood cancer HPB‐ALL cells through the upregulation of the proapoptotic proteins BAX and BH3‐interacting domain death agonist (BID) and downregulation of BCL‐2 and BCL‐XL expression along with disruption of potential of mitochondrial membrane (ΔΨm) and the activation of caspase‐3 (Liu et al., 2006).

ORI can induce endoplasmic reticulum (ER) stress activation, thus activating apoptotic signals regulating the kinase (ASK1)‐1 c‐Jun N‐terminal kinase 1 (JNK1) signaling to cause apoptosis and G2/M cell cycle arrest (Cai et al., 2013). ORI also induces apoptosis (time and dose dependent) by inhibiting tyrosine kinase activity and downregulating the expression or phosphorylation of epidermal growth factor receptor (EGFR). ORI also significantly affects the localization of EGFR and phosphorylated EGFR on the cell membrane (Li et al., 2007b). Furthermore, ORI has shown to induce significant growth and colony‐forming inhibition, cell cycle arrest at G2 to M phase transition, and apoptosis in dose‐dependent manner by increasing histone (H3 and H4) hyperacetylation, by activating p16, 21, and p27, and by suppressing the expression of cellular myelocytomatosis (c‐myc) oncogene (Gao, Hu, et al., 2010).

Diterpenoids derived from *I. rubescens* provide a novel method for inhibiting NF‐κβ DNA‐binding activity. Such substances interact with the p65 and p50 subunits of NF‐κβ at a location other than the DNA binding site. Thereby, the transcription factor's affinity for DNA is then modulated by distinct NF‐κβ binding sequences. Conclusively, the diterpenoid structure could be used as a scaffold to build more effective and selective NF‐κβ inhibitors that target regulated gene transcription (Leung et al., 2005).

Other studies demonstrated that ORI induces the activation of cellular tumor antigen p53 (p53) (that promotes the upregulation of a set of proteins associated with the cell cycle arrest at G2 to M phase and apoptosis). It also brings about the cleavage of p53‐induced E3 ubiquitin protein ligase Mdm2 (Msm2) by activating the caspases. The Mdm2‐p60 production after cleavage leads p53 accumulation and promotes its continuous activity in the cell. ORI‐mediated reactivation of p52 also causes the generation of ROS that further stimulates the cell apoptosis (Zhu et al., 2019).

Similarly, ORI also targets the expression of cyclinB1 and cyclin‐dependent kinase 2 whose downregulation along with the upregulation of p53 and p21 leads to the cell cycle arrest. This event further triggers the cleavage activation of caspase‐3, ‐8, and ‐9 as well as increasing the BAX to BCL‐2 ratio, which eventually leads to the apoptosome formation and the cell death (Jiang et al., 2019). ORI also induced apoptosis by promoting the truncation of BID and targeting the expression MCL1 protein (Han et al., 2020).

Ferroptosis is a type of cell death that is characterized by the accumulation of iron and the production of ROS in cells. It is a regulated form of cell death, and accumulation of iron and ROS in cells during ferroptosis is thought to be due to the activation of enzymes called iron‐dependent lipid peroxidases, which can cause the oxidation of certain types of lipids in the cell membrane (Hassannia et al., 2019).

Studies have shown that ORI can induce ferroptosis in cancer cells (Chen et al., 2021; Hassannia et al., 2019). It has been observed to inhibit the gamma‐glutamyl cycle in TE1 cells by causing significant changes in the activities of 5‐oxoproline, gamma‐glutamyl amino acids, glutamate, glutathione disulfide, and reduced glutathione. Furthermore, ORI has been found to inhibit the activity of glutathione peroxidase 4 (GPX4), an enzyme which is involved in the defense against oxidative stress and the prevention of ferroptosis. It can bind to cysteine for inhibiting the glutathione synthesis. By inhibiting GPX4 and gamma‐glutamyl cycle, ORI can induce ferroptosis to exert anticancer activity (Zhang et al., 2021).

### 5.2 Antiproliferation influence

ORI has been shown to suppress cancer cell proliferation in a concentration‐ and time‐dependent manner. Mechanistically, ORI can decrease the concentration of various Smad proteins (2, 3, 4) and plasminogen activator inhibitor 1. Furthermore, it downregulates the phosphorylation of only two Smads 2 and 3 brought on by transforming growth factor‐1 (TGF‐1). ORI administration inhibited tumor development in nude mouse model through the decrease in the expression of Smad proteins, pSmad 2 and pSmad 3 (Bu et al., 2019).

### 5.3 Inhibition of cancer cell invasion and metastasis

ORI also has potential to inhibit the metastatic spread and cell proliferation of cancer cells in a dosage‐dependent manner. By reducing the expression levels of the proteins, hypoxia‐inducible factor 1 (HIF‐1) and matrix metallopeptidase (MMP)‐9, ORI might block the epithelial‐to‐mesenchymal transition (EMT) due to hypoxia. In a GBC‐SD cell xenograft model, ORI also inhibited cell growth and reduced HIF‐1 and MMP‐9 expression levels. Notably, ORI targets the HIF‐1/MMP‐9 signaling pathway to inhibit tumor cell motility and EMT (Chen et al., 2019).

Similarly, sorafenib and ORI when combined, synergistically suppressed proliferation, invasion, migration, and EMT while inducing apoptosis by altering the AKT pathway without affecting NF‐κβ or MAPK signaling (Chen et al., 2019).

Likewise, ORI inhibited T24 cells' ability to proliferate, form colonies, and migrate by dramatically upregulating p53 and cleaved caspase‐3 expression levels while downregulating transient receptor potential cation channel subfamily M member 7, phosphorylated AKT (p‐AKT), and phosphorylation of ERK (p‐ERK) expression (Che et al., 2021).

### 5.4 Regulation of autophagy

Autophagy is a crucial metabolic process that helps to even out energy supply during critical stages in growth and in the face of nutrient shortages. It was noticed that, after ORI treatment the quantity of LC3B II protein was increased and the concentration of p62 and Beclin‐1 was also raised in malignant cells. This simultaneous restriction of the phosphoinositide 3‐kinase (PI3Ks) pathway and the stimulation of the c‐JNK pathway were responsible of the cancer cell proliferation, death, and autophagy processes after ORI treatment (Che et al., 2021).

## 6 ORI ACTIVITY IN DIFFERENT CANCERS IN VITRO STUDIES

Extensive research studies have been performed on different cancerous cell lines to observe the prominent impact of ORI on liver (Zhang et al., 2006), lung (Xu et al., 2020), gastric (Ren et al., 2020), CRC (Yao et al., 2017), breast (Wang et al., 2013), prostate (Lu et al., 2017), esophageal (Jiang et al., 2019), and other cancers through different pathways. ORI can induce retardation of the cancer growth and apoptosis of human cancer cells by promoting p53 expression and activity (Ikezoe et al., 2003). ORI also modulates the PI3K/AKT pathway, TGFβ pathway, and cell death pathways to exert its anticancer influence (Bu et al., 2019; Che et al., 2021; Han et al., 2020). The mechanisms employed by ORI to curb different cancers are enlisted in Table 2.

**TABLE 2:** Mechanism of action of ORI in different cancers.

Cancer	Anticancer effect	Dosage	Pathway	Reference
Liver cancer	Cell cycle arrest, apoptosis	41.8, 31.6, and 23.7 mmol/L	BCL‐2 and BAX	Liu et al. (2006)
Lung cancer	Apoptosis, antiproliferative effect	>28 μmol/L	BCL‐2 and BAX, ERK‐p53	Liu et al. (2004)
Colorectal cancer	Apoptosis		P16, p21, MAP kinase pathway	Jin et al. (2011)
Breast cancer	Autophagy, apoptosis		P53, p21, and mitochondrial pathways, β1/FAK pathway	Cui et al. (2007), Wang et al. (2013)
Esophageal cancer	Apoptosis		MDM2, p53, Bcl‐2 pathways, and caspase activation	Liu, Bu, et al. (2014), Wang et al. (2015)
Prostate cancer	Autophagy and apoptosis		P21 upregulation	Ye, Li, et al. (2012)
Skin cancer	Apoptosis, antiproliferation		BCL‐2, FGFR3, CCND1 pathways	Zhao et al. (2012)
Blood cancer	Phagocytic activity, cellular apoptosis and necrosis		ERK/NF‐κβ, Ras–Raf, PI3K‐Akt pathway	Liu, You, et al. (2005), Zang et al. (2012)
Epithelial cancer	Apoptosis and autophagy		EGFR, RAS/RAF/ERK signaling	Li et al. (2007a), Yu et al. (2012)
Bone cancer	Apoptosis, cell cycle arrest		MAPK pathway, BAX/BCL‐2 expression, NO‐ERK‐p53 pathway	Cheng, Qiu, and Ikejima (2009), Ye, Wang, et al. (2012)
Pancreatic and gallbladder cancer	Apoptosis and inflammation		JNK and p38 pathways, MAPK, p53	Wang, Wang, et al. (2014)
Laryngeal cancer	Apoptosis		EGFR signaling, p21/WAF‐1 pathway	Kang, Zhang, Qiu, Chen, et al. (2010)
Brain cancer	Apoptotic activity		Mitochondrial pathways	Lin et al. (2015), Yin et al. (2012)

Abbreviations: Bcl‐2, B‐cell lymphoma 2; BAX, Bcl‐2‐associated X protein; EGFR, Epidermal growth factor receptor; ERK, extracellular signal‐regulated kinase; p53, tumor protein p53; JNK, Jun N‐terminal kinase; MAPK, mitogen‐activated protein kinase; β1/FAK, integrin‐focal adhesion kinase; MDM2, murine double minute 2; FGFR3, fibroblast growth factor receptor 3; NF‐κβ, Nuclear factor kappa B; PI3K, phosphoinositide‐3‐kinase protein kinase; RAF, rapidly accelerated fibrosarcoma.

### 6.1 Liver cancer

In HCC (BEL‐7402), ORI downregulated and upregulated the expression of BCL‐2 and BAX expression, respectively. Furthermore, it also reduced the expression of telomerase reverse transcriptase mRNA along with telomerase activity (Liu et al., 2006). Investigations reported that ORI induces phase G2/M cell cycle arrest and apoptosis in human hepatoma (HepG2) cells through the alteration of MAPK and p53 metabolic pathways (Wang et al., 2010). Another study indicated that upregulation of Glycyl‐tRNA synthetase and heterochromatin protein 1 beta might facilitate inhibitory effects of ORI on tyrosine kinase and telomerase, respectively, in HepG2 cells (Wang, Ye, Pan, et al., 2011). α‐CP1 and ER stress had significant roles in promoting anticancerous effects of ORI in HepG2 cells, as revealed by proteomic and functional analyses (Wang, Ye, Chu, et al., 2011). It was also observed that ORI activates oxidative stress pathways in HepG2 cells to exhibit anticancer activity through the involvement of Hsp70‐1, Sti1, and Prdx2 OS markers (Wang, Ye, & Yu, 2014).

Galactose‐decorated pH‐responsive nanogels have the potential to increase the uptake of ORI in the human hepatoma HepG2 cells through endocytosis facilitated by asialoglycoprotein receptor. In liver cancer cells, targeted drug delivery can be well accomplished via these nanogels (Feng et al., 2011).

A study stated that ORI‐N significantly hindered the proliferation of HCC SMMC‐7721 cells by causing an arrest at the G2/M phase of cell cycle followed by increased apoptotic rate compared to the bulk ORI solution (Lou et al., 2011).

Researchers found that ORI helps in sensitization of hepatic cancer cells for optimal arsenic oxide therapy in HCC patients and acts synergistically with arsenic oxide (As_2_O_3_) to curb cancer growth (Zhao et al., 2012). ORI also initiated growth retardation and apoptotic pathways in cultured human hepatoblastoma HuH‐6 cells by stimulating ER stress and ASK1/JNK signaling pathways (Cai et al., 2013). In MHCC97‐H, the metastatic HCC cells, ORI stimulated apoptosis by reducing mitochondrial membrane potential (ΔΨm), followed by cytochrome C (CytC) and caspase‐3 and ‐9 activation (Zhu et al., 2013).

For the efficient liver targeting the delivery of ORI, ORI‐loaded galactosylated bovine serum albumin nanoparticles were developed which could enhance drug plasma levels as well as increase the circulation time as compared to ORI solution (Li, Zhang, et al., 2014a).

ORI may help inhibit the proliferation and fibrogenesis of hepatic stellate cells as it stimulates cell cycle arrest and cancerous cell apoptosis through p53–p21 pathways and does not cause toxicity in hepatocytes (Bohanon et al., 2014).

Computational research by Gao et al. revealed 102 differentially expressed genes (DEGs) shared by 790 and 2162 DEGs from the GSE25097 and TCGA LIHC datasets. Screening these common DEGs produced 22 hub genes, 16 of which had area under ROC curve >90%. The liver cancer survival rate was linked to ESR1, SPP1, and FOSB gene expression. Study reported that AURKA, SPP1, NUSAP1, UBE2C, TOP2A, AFP, PTTG1, GMNN, RRM2, CXCL12, SPARCL1, FOS, SOCS3, DCN, PCK1, and FOSB can be used as diagnostic biomarkers for liver cancer, among which FOBS and SPP1 genes can also be used as prognostic biomarkers (Gao et al., 2021; Gao et al., 2023).

### 6.2 Lung cancer

A study conducted on lung cancer SPC‐A‐1 cells indicated that ORI down‐ and upregulated the levels of BCL‐2 and BAX proteins to exhibit apoptosis and antiproliferative effects (Liu et al., 2004).

Another study revealed that ORI significantly increased the apoptotic pathways, specifically the c‐Met‐Bcl‐2‐Casp‐3 and c‐met‐ NF‐κβ‐COX‐2 pathways, in hepatocyte growth factor (HGF)‐stimulated A549 cells (Liu et al., 2012). The HGF inhibition augmented ERK‐p53 pathway activation, promoting autophagy and the mitochondrial membrane potential loss, resulting in apoptosis (Liu et al., 2013). A study conducted on A549 cells and normal human fetal lung fibroblast cell linings (MRC‐5) showed that ORI increased the antitumor activity of lentinan, making them a potential combination for treating lung cancer (Gui et al., 2021). Another study has revealed that ORI helps natural killer cells to express their cytotoxic effects against A549 lung cancer cells. It upregulates the expression of interferon gamma (IFN‐γ) and inhibits signal transducer and activator of transcription 3 (STAT3) phosphorylation in NK‐92MI cells (Hwang & Chang, 2023).

### 6.3 Gastric cancer

ORI remarkably inhibits the proliferation of gastric tumor and induces apoptosis by activating caspase pathway through upregulating BAX expression and changing BCL/BAX ratio (He et al., 2009). In ORI‐treated HGC‐27 cell line, differential expression of CytC, caspase‐3, and apoptosis protease‐activating factor‐1 is associated with the apoptosis (Sun et al., 2012). ORI may inhibit SGC‐7901 gastric cancer cells proliferation at IC_50_ 15.6 via modulating the TNF‐α/AR/TGF‐β signaling pathway axis (Gao et al., 2023). It also induces G2/M‐phase cell cycle arrest by decreasing the expression of cyclinB1 and CDK1 proteins (Gao et al., 2014). The anticancer influence of ORI is dosage‐ and time‐dependent pattern (Wang et al., 2021).

### 6.4 Colorectal cancer

ORI induces histone protein hyperacetylation and regulates the expression of c‐myc, p16, p21, and p27 to eventually cause senescence and apoptosis of CRC cells, making ORI a potent anti‐CRC compound (Gao, Hu, et al., 2010). ORI also downregulates the expression of the AP‐1 gene, followed by halted expression of NF‐κβ as well as MAPK pathways, ultimately ceasing tumor proliferation (Jin et al., 2011). ORI also induces reduction in thioredoxin reductase (TrxR) activity, a novel cancer target, which results in increased depletion of H_2_O_2_ and glutathione in cancer cells (Gao et al., 2012). In human CRC cells, the ORI anticancer effect is regulated by inhibiting the expression of SREBP1 mRNA and protein and fatty acid synthase (FAS) (Kwan et al., 2013).

ORI concentration‐dependent influence in human CRC LoVo and HCT‐116 cell cells is also reported. It suppresses miR‐32 expression, downregulates BCL‐2 expression, upregulates BAX, Bim, and cytosolic CytC expression, promotes cleavage of caspase‐3 and ‐9 proteins, halts the cancer proliferation, and stimulates apoptosis (Yang et al., 2015).

A recent study showed that ORI effectively suppressed the Warburg effect in cancer cells by downregulating dimeric pyruvate kinase M2 (PKM2), thereby preventing its nuclear translocation. Importantly, ORI exerted no significant influence on the EGFR/ERK signaling cascade, while exhibiting a notable reduction in the binding affinity between Importin‐α5 and the PKM2 dimer (Chen et al., 2023).

### 6.5 Breast cancer

In a study on human breast cancer (BC) cells, ORI concurrently stimulated both autophagy and apoptosis. In ORI‐treated MCF‐7 cells, ORI decreased the growth of cancerous cells and induced apoptosis through changes in caspase‐9 and mitochondrial pathways along with increased p53 and p21 expressions, causing cell cycle arrest, independent of caspase‐3 activity (Cui et al., 2007). Another study on the same cancer cells explained that ORI‐N more significantly retarded tumor proliferation by causing G2/M‐phase cell cycle arrest and induced apoptosis through BCL‐2/BAX pathway in a concentration‐dependent way, when compared with ORI solution (Feng et al., 2011). ORI also markedly caused DNA damage, resulting in cell cycle arrest through the activation of ataxia telangiectasia mutated (ATM) protein kinase which, in turn, activated checkpoint kinase 2 (CHK2) (Zhang, Tan, et al., 2013).

ORI stimulated apoptosis and inhibited metastasis of MDA‐MB‐231 cells through mediating the integrin β1/FAK pathway and reducing the ΔΨm (Wang et al., 2013). Another study showed that ORI phosphate (ORI derivative) suppressed MDA‐MB‐231 and MDA‐MB‐436 cells proliferation by time‐ and dose dependently increasing LC3‐II and beclin‐1 expression along with increased caspase‐9 activation and BCL‐2/BAX pathway regulation (Li et al., 2015). A recent study has further confirmed that ORI could inhibit the migration of breast cancer cells by arresting cells in S‐phase and affecting different cell cycle‐related proteins, including some kinases, p53, and p21 that ultimately hinders PI3K/AKT/mTOR signaling pathways (Zhang et al., 2023).

Analysis of 4T1 cells identified a total of 33 differential metabolites, including multiple amino acids (such as l‐glutamic acid, l‐asparagine, l‐histidine, l‐valine, and l‐isoleucine).

The novel dienone analogs of ORI having the A ring with an additionally installed α, β‐unsaturated ketone system exhibited significant apoptotic induction and growth retarding effects in superaggressive and drug‐resistant BC cells. Meanwhile, these analogs showed minimal toxic effects in normal human mammary epithelial cells, when compared to ORI (Ding et al., 2013b). In another study, among the analogs of ORI synthesized by removing multiple –OH groups and simplification of molecular structure, compound 56 turned out to be a competent candidate for treating triple‐negative breast cancer (Yao et al., 2020).

### 6.6 Esophageal cancer

ORI is found to suppress tumor proliferation and bring about apoptosis in the human esophageal squamous cell carcinoma (ESCC) cells EC9706 in time‐ and dosage‐dependent manner at range 10–40 μmol/L (Liu, Bu, et al., 2014).

In a recent study, researchers used a synergistic combination of ORI and cisplatin (CIS) against different ESCC cell lines, observing their mutual anticancer effects against ESCC. Results suggested that ORI can synergistically improve the antitumor effect of CIS, with p53 mutation and glutathione deficiency as biomarkers for the use of ORI and CIS in combination (Yang et al., 2022).

### 6.7 Prostate cancer

ORI and ponicidin (diterpenoids) exhibited prominent antiangiogenic activity at noncytotoxic concentrations, indicating them to be potent compounds in enhancing the clinical effectiveness of PC‐SPES, and can be used as a therapeutic herbal formulation for advanced prostate malignancy (Meade‐Tollin et al., 2004).

ORI stimulated autophagy in PC‐3 cells by increasing the expression of beclin1 mRNA level and MAP‐LC3, resulting in growth inhibition of PC cells (Ye et al., 2010). Another study demonstrated a significant improvement in ORI antiproliferative activity when used as ORI‐N which suggested it as a potent treatment for androgen‐independent prostate cancer (Zhang et al., 2010). Studies reported that ORI caused autophagy and apoptosis in PC‐3 and LNCaP cell lines through upregulation of p21 gene (Li, Wang, Ye, & Li, 2012; Ye, Li, et al., 2012).

### 6.8 Skin cancer

ORI induced human melanoma A375‐S2 cells apoptosis through activation of BAX‐mediated caspase pathway by stimulating the Cyt C release and caspase‐9 activation in the mitochondrial pathway (Zhang et al., 2004a). However, a study indicated that most ORI‐treated A375‐S2 cells (137.4 μmol/L for 12 h) also underwent necrosis, suggesting that ORI might cause A375‐S2 cell death by balancing apoptosis and necrosis rates.

A study conducted on murine melanoma K1735M2 cell line indicated that ORI induced growth retardation of cancerous cells through G2/M‐phase proliferative arrest and structural differentiation, stimulating K1735M2 cells to generate structures such as dendrites. This experiment suggested ORI as a potential therapeutic for melanoma cancer therapy (Ren et al., 2006).

ORI was also reported to simultaneously induce autophagy and cellular apoptosis by mediating the intracellular ROS generation and expression of SIRT1 nuclear protein in a time‐dependent pattern (Zeng et al., 2012). ORI also enhances the multiple human myeloma (MM) LP‐1 cellular apoptosis by upregulating the expression of Bid mRNA and downregulating the expression of BCL‐2, Myc, FGFR3, and CCND1, and decreasing the mitochondrial membrane potential ΔΨm. ORI also blocks the activation of NF‐κβ for apoptosis induction and cell proliferation inhibition (Duan et al., 2014; Zhao et al., 2012).

### 6.9 Association with blood cancer

ORI can increase the phagocytic activity against UV‐irradiated apoptotic human histocytic lymphoma U937 cells by stimulating the release of TNF‐α and IL‐1β, the potential contributors to ORI antitumor activities (Liu, You, et al., 2005). The feedback regulation of ERK/NF‐κβ/caspase‐1 may cause the release of IL‐1β (Zang et al., 2012). It was also found that NO improved efferocytosis of U937 cells, induced by ORI, via NF‐κβ‐COX‐2‐IL‐1β pathway activation (Zang et al., 2012). Hydrogen peroxide and hydroxyl ions also enhance ORI‐initiated phagocytosis in U937 cells via stimulation of PLCγ‐Ras‐Raf‐ERK and PI3K‐Akt signaling pathways, ΔΨm downregulation, and initiation of autophagy (Zang et al., 2013).

ORI remarkably induced apoptosis and antiproliferative effects on NB4 cells by caspase‐3 activation and poly‐ADP ribose polymerase cleavage along with BCL‐2 downplay and ΔΨm disruption. These findings suggested that ORI can be used in a potential antileukemia therapy (Liu, Huang, et al., 2005). Similar results were found in a study on HPB‐ALL cells where ORI induced antiproliferative effects through cellular apoptosis and necrosis by disrupting ΔΨm, activating caspase‐3, and regulating BCL/BAX pathway (Liu et al., 2006).

A study revealed that ORI stimulated t(8;21) acute myeloid leukemia (AML) cell line apoptosis along with the degradation of AML1‐ETO (AE) fusion oncoprotein by targeting its D188 residue, with minimal side effects (Zhou et al., 2007).

The potential of ORI on ATRA (all‐trans‐retinoic acid) sensitive and resistant APL cells, namely NB4 and NB4‐R1 cancerous cells, respectively, was also determined. A combinatory treatment of ATRA and ORI caused cellular differentiation and promoted ROS stimulated apoptosis (Gao, Tang, et al., 2010). ORI also increased cellular ROS levels to stabilize retinoic acid receptor alpha (RARα) protein, which in turn activated NF‐κβ signaling pathway. This whole mechanism helps prevent RARα activity malfunctioning, which can otherwise lead to the pathogenesis of acute promyelocytic leukemia (Cao et al., 2015). ORI also stimulates cytosolic entry of Ca^2+^ and formation of ceramide in erythrocytes that initiates eryptosis (Jilani et al., 2011).

The potential of ORI with imatinib was reported on Ph(+) acute lymphoblastic leukemia cells where ORI hindered LYN/mTOR signaling pathway activation and indicated a mutual antileukemia effect with imatinib (Guo et al., 2012b). Furthermore, ORI impeded activation of ABL kinase along with RAF/MEK/ERK, Akt/mTOR, and STAT5 signaling pathways, and regulated BCL‐2/BAX expression to exhibit antileukemia effect on Ph(+) ALL SUP‐B15 cells (Guo et al., 2012a).

In human erythroleukemia OCIM2 cells, ORI stimulated apoptosis through activation of NF‐κβ and downregulation of mRNA and protein levels of nucleoporin 214 and nucleoporin 88 (Yi et al., 2012).

In Jurkat cells, ORI targets multifunctional, stress‐inducible heat shock protein 70 1A (HSP70 1A). In NPM1c+ AML cells, ORI caused activation of caspase‐3, translocation NPM1 mutant protein (NPM1c+) into the nucleus via nuclear accumulation of Exportin 1 and apoptosis (Dal Piaz et al., 2013). Moreover, ORI significantly increased the expressions of p14arf and p53 proteins, contributing to cellular apoptosis (Li, Yi, et al., 2014).

An important investigation stated that cancer cell chemoresistance was observed to be reversed by ORI. Treatment of antileukemia drugs (VP‐16 and Ara‐C) resistance human leukemia cells HL60, K562/ADR, and K562 with ORI resulted in the apoptosis induction. ORI stimulated mitochondria‐dependent apoptosis by de‐repressing BIM‐S by inhibiting miR‐17 and miR‐20a expressions (Weng et al., 2014).

ORI exerted an antileukemia effect in human T‐cell ALL cell line CEM by halting the stimulation of mTOR/P70S6/4EBP1, RAF/ERK, and STAT5 signaling pathways, downregulation of BCL‐2 expression, and upregulation of BAX expression (Yong et al., 2014).

### 6.10 Epithelial cancer cell lines

Study revealed that ORI induced apoptosis in human epidermoid carcinoma A431 cell by blocking EGFR and RAS/RAF/ERK signaling pathways (Li et al., 2007a). ORI also inhibited tyrosine kinase activity and EGFR tyrosine phosphorylation to induce apoptosis in A431 cells (Li et al., 2007b). Another study stated that ORI stimulated caspase‐3 activation, TK activity inhibition, and mitochondrial pathway‐dependent apoptosis (Li et al., 2008). Hydroxyl radical also facilitates ORI‐induced autophagy and apoptosis (Yu et al., 2012).

Combined ORI and γ‐tocotrienol therapy showed synergistic anticancer activity on mouse +SA MEC by the induction of autophagy through the expression of ATG factors, beclin‐1, LAMP‐1, and cathepsin‐D as well as LC3B‐I to LC3B‐II conversion and AKT/mTOR pathway suppression (Tiwari et al., 2015).

### 6.11 Cancers of bone and connective tissue

In a study, ORI with cisplatin synergistically showed enhanced cytotoxicity and DNA cross‐linking in murine sarcoma S180 cells than cisplatin alone (Gao et al., 1993). ORI induced mitochondria‐ and MAPK‐dependent apoptosis in the murine fibrosarcoma L929 cells by augmenting the ratio of BAX/BCL‐2 protein expression (Zhang et al., 2004b). ORI also increased the expression of pro‐TNF‐α as well as IkB phosphorylation to cause apoptotic cellular death (Huang et al., 2005). Apoptosis induced by ORI also involved ROS‐regulated signaling pathways. However, ORI‐stimulated autophagy inhibited apoptosis in L929 by increased activation of p38 and NF‐κβ (Cheng, Qiu, & Ikejima, 2009). Moreover, ORI caused G2/M‐phase arrest followed by apoptosis through ERK‐p53 pathway activation and PTK‐regulated survival pathway inhibition (Cheng, Qiu, Ye, et al., 2009). NO also plays a significant role in ORI‐initiated apoptotic mechanism through NO‐ERK‐p53 pathway (Ye, Li, et al., 2012).

ORI can enhance apoptosis in human osteosarcoma (OS) cells via mitochondrial and caspase pathway by deactivating ERK and AKT, and activating signaling pathways involving JNK and p38 MAPK pathways (Jin et al., 2007). Furthermore, ORI also inhibits the Wnt/β‐catenin signaling transduction through augmenting Dkk‐1 expression and GSK3β functionality (Liu, et al., 2014).

### 6.12 Pancreatic and gallbladder cancers

ORI effectively caused G2/M stage cycle arrest followed by induction of apoptotic pathways in pancreatic cancerous cell linings (PANC‐1) (Qi et al., 2012). Treatment with 80 μmol/L of ORI downregulated protein expression of JNK and p38, and increased the expression of p‐JNK and p‐p38 (Wang, Wang, et al., 2014). Pancreatic cancer cells SW1990 underwent ORI‐initiated apoptosis via p38 MAPK pathway inhibition and induction of p53‐ and caspase‐dependent signaling mechanisms (Bu et al., 2014).

ORI can potentiate the anticancerous potential of gemcitabine drug in pancreatic cancer by activation of p53 that mediates MAPK‐p38 signaling pathway (Bu et al., 2012). Through the BCL‐2/BAX mechanism, ORI induced mitochondrial pathway‐dependent apoptotic death in gemcitabine‐treated pancreatic cancer cells (Liu, Bu, et al., 2014).

ORI caused hallmark changes on inflammatory pathways as it decreased IL‐1β, IL‐6, and IL‐33 expressions in human pancreatic cancer BxPC‐3 cells by regulating the expression of various nuclear transcription factors in a dosage‐dependent pattern (Chen et al., 2014). On the same cancer cell line, ORI treatment in low‐dose mediated p53 and CDK1 expression to hinder cellular proliferation via G2/M‐phase arrest. On the other hand, ORI treatment in high dose caused S‐phase cellular accumulation as a result of DNA damage (Xu et al., 2015). ORI also modified the expression status of microRNAs that exhibit antitumor effects in BxPC‐3 cells (Gui et al., 2015).

ORI is found to induce prominent growth retardation, S‐phase cell cycle arrest, and apoptotic cell death in human gallbladder carcinoma SGC996 and NOZ cells via mitochondria‐dependent pathway (Bao et al., 2014).

### 6.13 Laryngeal cancer

ORI inhibited EGFR signaling that increased apoptotic rate in human laryngeal cancerous cells by increasing oxidative stress accompanied by the stimulation of extrinsic and intrinsic apoptotic pathways (Kang, Zhang, Qiu, Tashiro, et al., 2010). ORI stimulated ROS generation and G2/M cycle arrest in Hep‐2 cell line, resulting in p21/WAF1‐dependent apoptosis (Kang, Zhang, Qiu, Chen, et al., 2010). ORI also reduced the expression of caspase‐9 by altering the production of ROS and activating ER stress that initiated apoptosis progression (Kang et al., 2015; Kou et al., 2020).

### 6.14 Brain/spinal cord and nerve cancers

ORI suppresses the proliferation and initiates the process of cell death in astrocytoma cells via mitochondrial pathway (Yin et al., 2012). Similar findings were indicated in both human glioma U87 and U251 cells that underwent S‐phase arrest due to ORI, leading to apoptosis (Zhang, Bao, et al., 2014; Zhang, Liu, et al., 2014). In U87MG human glioma cell, ORI caused decrease in the cytoplasmic concentration of RanGTPase activating protein 1 (RanGAP1) that led to the accumulation of RanGTP and RNA in the nucleus and induced U87MG cell death (Lin et al., 2015). ORI in combination with NVP‐BEZ235 extraordinarily incited apoptosis of neuroblastoma cells. Prominently, the synergistic enactment of the apoptotic pathway was going with upgraded autophagy as proven by critical diminished p62 articulation as well as upregulated transformation of LC3‐II (Ding et al., 2016).

### 6.15 Other cancer cell lines

ORI and its various derivatives have shown significant antiproliferative, apoptotic, and autophagy activities in various cancer cell lines, such as NO‐releasing novel furozan/ORI hybrids (Li, Wang, Cai, et al., 2012), enmein‐type 14‐O‐diterpenoid derivatives (Li et al., 2013), derivatives with thiazole‐fused A‐ring (Ding et al., 2013a, 2013b), and others (Xu et al., 2014).

In cervical carcinoma HeLa cell line, ORI hindered the cellular proliferation and stimulated apoptosis through PI3K/Akt pathway by inhibiting PI3K targets namely FOXO, AKT, and GSK3 (Hu et al., 2007). ROS are significantly involved in ORI‐stimulated apoptosis and autophagy (Zhang et al., 2011). ORI also showed synergistic antiproliferation action and apoptosis induction with wogonin in many epithelial ovarian cancer cell lines (Chen et al., 2011).

ORI displayed declined cell viability, initiated apoptosis, and hindered clonogenic survival and growth in human uveal melanoma MUM2B and OCM‐1 cell lines through upregulation of Bim (BCL2‐interacting mediator of cell death) and inhibition of FAS (Gu et al., 2015).

A study on thyroid cancer cells (TCC) indicated ORI targeting JAK2 for deactivating JAK2/STAT3 pathway to ultimately suppress angiogenesis and epithelial–mesenchymal transition of TCC (Liu et al., 2022).

## 7 IN VIVO STUDIES

A study showed remarkable antitumor effects of ORI and other kaurane‐type diterpenoids by their intraperitoneal injection to the test mice with inoculated Ehrlich ascites carcinoma. Later, the activity of both compounds was observed and stated in terms of their structural feature (Fujita et al., 1976). ORI's in vivo activity is mainly reported for gastric cancer and prostate cancer that are discussed next.

### 7.1 Prostate cancer

PC‐SPES, a herbal medication used for treating prostate cancer, had two marker compounds namely baicalin and ORI, both exhibited antiproliferative potentials in prostate cancer cells, perhaps through downregulation of androgen receptor as well as initiation of apoptosis through increased p53 expression and inhibition of BCL‐2 gene (Marks et al., 2002).

### 7.2 Gastric cancer

ORI injection instigated apoptosis and effectively suppressed the growth of heterotransplanted human gastric adenocarcinoma in nude mice and A549 and NCI‐H292 xenografted cells (Chen et al., 2008; Wang & Zhu, 2019). Mechanistically, ORI modulates the activity of mTORC1 and increases the concentration of BAX (Wang, Lv, et al., 2014). In a study, ORI caused antiproliferative and antiangiogenesis effects on human gastric cancer SNU‐5 subcutaneous xenograft model by directly mediating c‐Met signaling pathway (Liu, Qian, & Shen, 2014). Similarly, ORI obscures the signal transduction through Notch pathway to impede the tumor growth and metastasis in human umbilical vascular endothelial cells. Immunohistochemistry analysis indicated that ORI significantly dropped CD31 and vWF protein expressions in xenografts (Dong et al., 2014).

ORI stimulated cellular growth inhibition and apoptosis by regulating an E3 ligase–substrate pair (Fbw7‐c‐Myc) (Huang et al., 2012). However, to make the efficacy of ORI (or single therapeutic agent) long lasting, combined therapy that combines agents that target and degrade c‐Myc with microRNAs targeting agents might stand out as a novel treatment approach for cancer therapy (Huang et al., 2014). ORI also elicits autophagy significantly by negatively regulating RAS/PI3KCI/Akt pathway (Zhang et al., 2012).

## 8 POTENTIAL BIOTECHNOLOGICAL STUDIES ON IN VITRO CULTURES

Different biotechnological studies have explained the anticancer potential of ORI on other cultures. In a study, it was indicated that ORI‐loaded ORI‐PCL‐PEO‐PCL‐NPs increased the survival time of mice and showed more effectiveness compared with free ORI (Feng et al., 2008).

ORI (20 μg/mL) + LNT (200 μg/mL)‐treated SMMC‐7721 cells disclosed the most observable apoptotic rate, which was 40.5 ± 2.5%, which was greater than that of cells cured with LNT. ORI can act as a sharpening specialist to expand the anticancer movement of LNT in vivo. The past review has exhibited that ORI prompts cell apoptosis. Moreover, in vitro analysis, it sensitized liver disease cell lines to radiotherapy, and its apoptotic influences enhanced with synergistic treatment of LNT and ORI (Gao, Hu, et al., 2010).

Similarly, ORI encapsulation into RGD‐modified poly‐PLA nanoparticles as ORI‐PLA‐RGD‐NPs enhanced antitumor efficacy of ORI (Xu et al., 2012).

ORI is poorly water soluble. Therefore, researchers used ORI‐loaded MPEG‐PCL micelles as a drug carrier that maintained anticancer activity of ORI and showed significant potential for both direct IV and transdermal drug administration and delivery system in chemotherapy (Xue et al., 2012).

The impact of ORI on expansion was assessed by MTT examine, and cell relocation and attack were evaluated by trans well movement and intrusion tests in human bosom malignant growth cells. Furthermore, the statement of Notch receptors (Notch 1–4) was distinguished by western smear (Xia et al., 2017). ORI, a regularly utilized malignant growth chemotherapy drug, is adsorbed into PCN‐222 using the dissolvable dissemination procedure. In light of an examination of the ORI discharge profile, results propose that it can remain in the system for over 7 days in vitro (Leng et al., 2018).

Current anticancer medication advancement systems prevalently center around inhibitors of the particular atomic effectors associated with cancer cell multiplication (Yao et al., 2017).

In this review, we uncovered that the sharpening impact of ORI on tumor‐necrosis factor‐related apoptosis‐inducing ligand (TRAIL) prompted apoptotic pathways in a few disease cells. ORI improved passing flagging actuating buildings (Disk) arrangement and DR5 glycosylation without influencing the articulation of downstream intracellular apoptosis‐related proteins. Outcomes recommend that ORI‐actuated DR5 glycosylation adds to TRAIL‐prompted apoptotic cell passing in disease cells (Jeon et al., 2019).

The SARS‐CoV‐2 3C‐like protease (3CLpro), which is essential for the replication of virus and record, has been perceived as a perfect medication goal. A study revealed that three home remedies, salvianolic corrosive A, (−)‐epigallocatechin gallate, and ORI, straightforwardly hindered the movement of SARS‐CoV‐2 3CLpro. By tackling the design of 3Clpro in complex with ORI and contrasting it with that of different ligands with 3Clpro, it is distinguished that ORI ties at the 3Clpro reactant site by framing a C‐S covalent security, hindering substrate restricting through a nonpeptidomimetic covalent restricting mode. In this way, ORI is a potent candidate to present as another antiviral treatment for COVID‐19 (Jeon et al., 2019).

Bombesin receptor subtype‐3 (BRS‐3) is a vagrant G‐protein‐coupled receptor. Ongoing examinations have shown that BRS‐3 assumed an essential part in glucose guideline, insulin discharge, and energy homeostasis. Numerous cell‐based examines and in vivo analyses were performed to distinguish the new ligand. BRS‐3 overexpression cells were combined with FLIPR examine, homogeneous time‐settled fluorescence. IP‐ONE measure, dynamic mass reallocation examine, β‐arrestin2 enrolment examine, and western smudge to decide receptor initiation and downstream flagging occasions. ORI, as the found new ligand of BRS‐3, gives an important device compound to explore BRS‐3's capability, particularly for target approval in type 2 diabetes and corpulence. ORI is a promising lead compound in the treatment of metabolic problems (Zhu et al., 2022).

Another investigation assessed ORI properties for the hepatic oxidation, hostile to glycative and mitigating properties at 0.125% and 0.25% against ongoing ethanol consumption in mice. Ethanol stifled the hepatic mRNA articulation of atomic component E2‐related factor 2. ORI supplements were able to reverse the ethanol influence. Ethanol expanded the hepatic arrival of cancer rot factor‐alpha, changing development factor‐beta1, IL1β, and IL‐6. These clever discoveries propose that ORI could be utilized as a powerful specialist against liquor initiated hepatotoxicity (Yan et al., 2021).

Recently, ORI was incorporated into anisamide‐lipid calcium phosphate nanoparticles to synthesize AS‐ORI LCPs that resulted in stable drug release in acidic in vivo tumor environment, making it a potent drug formulation for treating lung cancer (Shen & Ma, 2022).

## 9 HUMAN CLINICAL STUDIES

Significant advancements have been made in ORI's structural optimization and mechanism of action studies over the past 10 years for the treatment of cancer and other disorders (Ding et al., 2016; Li, Han, Liao, et al., 2016; Owona & Schluesener, 2015). In addition to the changes on the A ring, the hydroxyl in the C ring can also be esterified to increase ORI's antiproliferative effect. For instance, HAO472 (Sun et al., 2014) was created with an alanine ester trifluoroacetate at the C‐14 position to increase its solubility in water (165 mg/mL), HAO472 was claimed to maintain ORI's anticancer properties (data not disclosed) while also being less likely to result in vascular injury (unavailable evidence) (Liu et al., 2016; Sun et al., 2014). HAO472 was recently approved for advancement into a Phase I human clinical trial (80–320 mg/day, i.v., CTR20150246) in China for the treatment of acute myelogenous leukemia. Hengrui Medicine Co. Ltd. (Lianyungang, China) received this approval.

### 9.1 Toxicity, side effects, and safety

The toxicity and safety of substances should be taken into account while evaluating their efficacy (Sun et al., 2020). ORI, a diterpenoids molecule found in many medicinal plants, has a wide spectrum of pharmacological properties. However, multiple lines of research suggested that ORI may have negative effects, even toxicity, under certain conditions, sparking heated debate and worry over ORI's safety. ORI was found to have anticancer effect against small cell lung cancer, however at the same time, HE staining exhibited some cytotoxicity in hepatic tissue following treatment with ORI (10 mg/kg) (Xu et al., 2020). At an EC50 of 411.94 mg/L in vitro, ORI treatment also caused abnormalities in zebrafish, including uninflated swim bladders and pericardial congestion, as well as a reduction in body length. The downregulation of vascular endothelial growth factor receptor 3 gene expression may be associated with the development of defects after exposure to ORI during embryonic development (Tian et al., 2019). According to a study, (Jilani et al., 2011), a 48‐h exposure to ORI (≥25 μM) dramatically increased the cytosolic Ca^2+^ concentration, potentiated ceramide synthesis, and eventually caused erythrocytes to commit suicide.

On the other hand, some reports claimed that ORI could induce the expression and activation of CYP2C and CYP3A family (Yi‐Wen et al., 2018) and they claimed that this could pose a risk to herb–drug interactions (Zhang, Bao, et al., 2014). Due to the safety concerns raised by the combination of ORI in clinical practice, these investigations suggested that we should pay attention to them.

Due to its exceptional anticancer activity, ORI has been the focus of growing interest among cancer biologists in recent years. ORI has relatively low toxicity while exerting broad anticancer effects on a variety of human cancer cells. The ORI's distinct and secure anticancer pharmacological profile sparked attention in the study of its anticancer action mechanism and structural alterations.

## 10 CONCLUSION

Several preclinical investigations have shown the anticancer effect of the plant‐derived chemical ORI. Breast, ovarian, and prostate cancer cell lines have all been shown to exhibit growth inhibition by it. Additionally, it has been shown that ORI induces apoptosis in cancer cells and has antiangiogenic action, which means it may prevent the growth of new blood vessels that tumors need to grow and spread. It affects diverse processes, such as the BCL‐2/BAX pathway, NF‐κβ, and p53‐mediated pathways, and others, which leads to antiproliferative, antiangiogenesis, autophagy, and apoptotic effects. When incorporated into different nanoparticle suspensions and formulations, ORI becomes an effective tool for cancer therapy. Thus, further studies must be conducted on increasing ORI's bioavailability and enhancing its anticancer properties. However, it should be noted that despite in China ORI is prescribed as an herbal anti‐inflammatory alternative medicine, the Food and Drug Administration has not approved any ORI‐based medication to be used in the treatment of cancer or other disorders yet. Therefore, more clinical research focused on investigating the efficacy of ORI in the treatment of different forms of cancer, such as breast, lung, and pancreatic cancer are necessary. These studies need to have as their primary objectives the determination of the best way to take ORI, both in terms of dose and administration, as well as any possible side effects or drug interactions. The findings of these investigations could contribute to the development of novel cancer treatments that make use of ORI as coadjutant, which has the potential to enhance the quality of life for cancer patients.

## AUTHOR CONTRIBUTIONS

**Muhammad Asif Ali:** Data curation (equal); investigation (equal); writing – original draft (equal); writing – review and editing (equal). **Noohela Khan:** Data curation (equal); investigation (equal); writing – original draft (equal); writing – review and editing (equal). **Ahmad Ali:** Data curation (equal); investigation (equal); writing – original draft (equal); writing – review and editing (equal). **Hira Akram:** Data curation (equal); investigation (equal); writing – original draft (equal); writing – review and editing (equal). **Noushaba Zafar:** Data curation (equal); investigation (equal); writing – original draft (equal); writing – review and editing (equal). **Kinza Imran:** Data curation (equal); investigation (equal); writing – original draft (equal); writing – review and editing (equal). **Tooba Khan:** Data curation (equal); investigation (equal); writing – original draft (equal); writing – review and editing (equal). **Khushbukhat Khan:** Data curation (equal); investigation (equal); methodology (equal); project administration (equal); validation (equal); writing – original draft (equal); writing – review and editing (equal). **Muhammad Armaghan:** Data curation (equal); investigation (equal); visualization (equal); writing – original draft (equal). **Marta Palma‐Morales:** Data curation (equal); investigation (equal); writing – original draft (equal); writing – review and editing (equal). **Celia Rodríguez‐Pérez:** Data curation (equal); investigation (equal); supervision (equal); validation (equal); writing – original draft (equal); writing – review and editing (equal). **Angela Caunii:** Data curation (equal); investigation (equal); writing – original draft (equal); writing – review and editing (equal). **Monica Butnariu:** Data curation (equal); investigation (equal); methodology (equal); validation (equal); visualization (equal); writing – original draft (equal); writing – review and editing (equal). **Solomon Habtemariam:** Data curation (equal); investigation (equal); project administration (equal); validation (equal); writing – original draft (equal); writing – review and editing (equal). **Javad Sharifi Rad:** Conceptualization (equal); data curation (equal); investigation (equal); methodology (equal); project administration (equal); supervision (equal); validation (equal); visualization (equal); writing – original draft (equal); writing – review and editing (equal).

## CONFLICT OF INTEREST STATEMENT

The authors have no relevant financial or other conflicts of interest.

## Data Availability

The authors offer all the data needed to support the study's findings in this article.

## References

[fsn33986-bib-0001] Bao, R. , Shu, Y. , Wu, X. , Weng, H. , Ding, Q. , Cao, Y. , Li, M. , Mu, J. , Wu, W. , Ding, Q. , & Tan, Z. (2014). Oridonin induces apoptosis and cell cycle arrest of gallbladder cancer cells via the mitochondrial pathway. BMC Cancer, 14(1), 1–13. 24655726 10.1186/1471-2407-14-217 PMC3994450

[fsn33986-bib-0002] Bohanon, F. J. , Wang, X. , Ding, C. , Ding, Y. , Radhakrishnan, G. L. , Rastellini, C. , Zhou, J. , & Radhakrishnan, R. S. (2014). Oridonin inhibits hepatic stellate cell proliferation and fibrogenesis. Journal of Surgical Research, 190(1), 55–63. 24742622 10.1016/j.jss.2014.03.036 PMC4225779

[fsn33986-bib-0003] Bu, H.‐Q. , Liu, D.‐L. , Wei, W.‐T. , Chen, L. , Huang, H. , Li, Y. , & Cui, J.‐H. (2014). Oridonin induces apoptosis in SW1990 pancreatic cancer cells via p53‐and caspase‐dependent induction of p38 MAPK. Oncology Reports, 31(2), 975–982. 24297112 10.3892/or.2013.2888

[fsn33986-bib-0004] Bu, H.‐Q. , Luo, J. , Chen, H. , Zhang, J.‐H. , Li, H.‐H. , Guo, H.‐C. , Wang, Z.‐H. , & Lin, S.‐Z. (2012). Oridonin enhances antitumor activity of gemcitabine in pancreatic cancer through MAPK‐p38 signaling pathway. International Journal of Oncology, 41(3), 949–958. 22710877 10.3892/ijo.2012.1519

[fsn33986-bib-0005] Bu, H.‐Q. , Shen, F. , & Cui, J. (2019). The inhibitory effect of oridonin on colon cancer was mediated by deactivation of TGF‐β1/Smads‐PAI‐1 signaling pathway *in vitro* and vivo. Oncotargets and Therapy, 12, 7467–7476. 31686852 10.2147/OTT.S220401 PMC6752205

[fsn33986-bib-0006] Butt, S. S. , Khan, K. , Badshah, Y. , Rafiq, M. , & Shabbir, M. (2021). Evaluation of pro‐apoptotic potential of taxifolin against liver cancer. PeerJ, 9, e11276. 34113483 10.7717/peerj.11276 PMC8162243

[fsn33986-bib-0007] Cai, D.‐T. , Jin, H. , Xiong, Q.‐X. , Liu, W.‐G. , Gao, Z.‐G. , Gu, G.‐X. , & Qiu, Y.‐H. (2013). ER stress and ASK1‐JNK activation contribute to oridonin‐induced apoptosis and growth inhibition in cultured human hepatoblastoma HuH‐6 cells. Molecular and Cellular Biochemistry, 379, 161–169. 23580093 10.1007/s11010-013-1638-2

[fsn33986-bib-0008] Cao, Y. , Wei, W. , Zhang, N. , Qing, Y. , Wen‐Bin, X. , Wen‐Jun, Y. , Chen, G.‐Q. , Ying‐Li, W. , & Yan, H. (2015). Oridonin stabilizes retinoic acid receptor alpha through ROS‐activated NF‐κB signaling. BMC Cancer, 15(1), 1–12. 25886043 10.1186/s12885-015-1219-8 PMC4403721

[fsn33986-bib-0009] Che, X. , Zhan, J. , Zhao, F. , Zhong, Z. , Chen, M. , Han, R. , & Wang, Y. (2021). Oridonin promotes apoptosis and restrains the viability and migration of bladder cancer by impeding TRPM7 expression via the ERK and AKT signaling pathways. BioMed Research International, 2021, 4340950. 34285910 10.1155/2021/4340950 PMC8275389

[fsn33986-bib-0010] Chen, F. , Liao, J. , Pinghui, W. , Cheng, L. , Ma, Y. , Zhang, L. , Leng, X. , Zhu, X. , Liu, Z. , & Xie, F. (2023). Oridonin inhibits the occurrence and development of colorectal cancer by reversing the Warburg effect via reducing PKM2 dimer formation and preventing its entry into the nucleus. European Journal of Pharmacology, 175856. 37321470 10.1016/j.ejphar.2023.175856

[fsn33986-bib-0011] Chen, J.‐H. , Wang, S.‐B. , Li, E.‐M. , Chen, L.‐M. , Yuan, S.‐J. , Wang, R.‐L. , & Shen, Z.‐Y. (2008). Inhibitory effect of Oridonin injection on heterotransplanted gastric adenocarcinoma in nude mice and its mechanism. Zhonghua Zhong Liu Za Zhi [Chinese Journal of Oncology], 30(2), 89–92. 18646687

[fsn33986-bib-0012] Chen, K. , Ye, J. , Qi, L. , Liao, Y. , Li, R. , Song, S. , Zhou, C. , Feng, R. , & Zhai, W. (2019). Oridonin inhibits hypoxia‐induced epithelial‐mesenchymal transition and cell migration by the hypoxia‐inducible factor‐1α/matrix metallopeptidase‐9 signal pathway in gallbladder cancer. Anti‐Cancer Drugs, 30(9), 925–932. 10.1097/cad.0000000000000797 31517732

[fsn33986-bib-0013] Chen, R.‐Y. , Xu, B. , Chen, S.‐F. , Chen, S.‐S. , Zhang, T. , Ren, J. , & Xu, J. (2014). Effect of oridonin‐mediated hallmark changes on inflammatory pathways in human pancreatic cancer (BxPC‐3) cells. World Journal of Gastroenterology: WJG, 20(40), 14895–14903. 25356049 10.3748/wjg.v20.i40.14895 PMC4209552

[fsn33986-bib-0014] Chen, S. , Cooper, M. , Jones, M. , Madhuri, T. K. , Wade, J. , Bachelor, A. , & Butler‐Manuel, S. (2011). Combined activity of oridonin and wogonin in advanced‐stage ovarian cancer cells: Sensitivity of ovarian cancer cells to phyto‐active chemicals. Cell Biology and Toxicology, 27, 133–147. 20872277 10.1007/s10565-010-9176-0

[fsn33986-bib-0015] Chen, X. , Dai, X. , Liu, Y. , He, X. , & Gong, G. (2022). *Isodon rubescens* (Hemls.) Hara.: A comprehensive review on traditional uses, phytochemistry, and pharmacological activities. Frontiers in Pharmacology, 13, 766581. 35401233 10.3389/fphar.2022.766581 PMC8987129

[fsn33986-bib-0016] Chen, X. , Kang, R. , Kroemer, G. , & Tang, D. (2021). Broadening horizons: The role of ferroptosis in cancer. Nature Reviews Clinical Oncology, 18(5), 280–296. 10.1038/s41571-020-00462-0 33514910

[fsn33986-bib-0017] Cheng, B. , Jin, J. , Liu, D. , Shi, T. , Xiaoqing, F. , Liu, C. , Liu, S. , & Rui, W. (2021). Oridonin interferes with simple steatosis of liver cells by regulating autophagy. Tissue and Cell, 72, 101532. 33823340 10.1016/j.tice.2021.101532

[fsn33986-bib-0018] Cheng, Y. , Qiu, F. , & Ikejima, T. (2009). Molecular mechanisms of oridonin‐induced apoptosis and autophagy in murine fibrosarcoma L929 cells. Autophagy, 5(3), 430–431. 19202353 10.4161/auto.5.3.7896

[fsn33986-bib-0019] Cheng, Y. , Qiu, F. , Ye, Y.‐C. , Tashiro, S.‐I. , Onodera, S. , & Ikejima, T. (2009). Oridonin induces G2/M arrest and apoptosis via activating ERK–p53 apoptotic pathway and inhibiting PTK–Ras–Raf–JNK survival pathway in murine fibrosarcoma L929 cells. Archives of Biochemistry and Biophysics, 490(1), 70–75. 19699177 10.1016/j.abb.2009.08.011

[fsn33986-bib-0020] Cui, Q. , Yu, J.‐H. , Wu, J.‐N. , Tashiro, S.‐I. , Onodera, S. , Minami, M. , & Ikejima, T. (2007). P53‐mediated cell cycle arrest and apoptosis through a caspase‐3‐independent, but caspase‐9‐dependent pathway in oridonin‐treated MCF‐7 human breast cancer cells. Acta Pharmacologica Sinica, 28(7), 1057–1066. 17588343 10.1111/j.1745-7254.2007.00588.x

[fsn33986-bib-0022] Ding, C. , Zhang, Y. , Chen, H. , Yang, Z. , Wild, C. , Chu, L. , Liu, H. , Shen, Q. , & Zhou, J. (2013). Novel nitrogen‐enriched oridonin analogues with thiazole‐fused A‐ring: Protecting group‐free synthesis, enhanced anticancer profile, and improved aqueous solubility. Journal of Medicinal Chemistry, 56(12), 5048–5058. 23746196 10.1021/jm400367n PMC3712786

[fsn33986-bib-0023] Ding, C. , Zhang, Y. , Chen, H. , Yang, Z. , Wild, C. , Ye, N. , Ester, C. D. , Xiong, A. , White, M. A. , Shen, Q. , & Zhou, J. (2013). Oridonin ring A‐based diverse constructions of enone functionality: Identification of novel dienone analogues effective for highly aggressive breast cancer by inducing apoptosis. Journal of Medicinal Chemistry, 56(21), 8814–8825. 24128046 10.1021/jm401248x PMC3880594

[fsn33986-bib-0024] Ding, Y. , Ding, C. , Ye, N. , Liu, Z. , Wold, E. A. , Chen, H. , Wild, C. , Shen, Q. , & Zhou, J. (2016). Discovery and development of natural product oridonin‐inspired anticancer agents. European Journal of Medicinal Chemistry, 122, 102–117. 27344488 10.1016/j.ejmech.2016.06.015 PMC5003635

[fsn33986-bib-0025] Ding, Y. , Li, D. , Ding, C. , Wang, P. , Liu, Z. , Wold, E. A. , Ye, N. , Chen, H. , White, M. A. , Shen, Q. , & Zhou, J. (2018). Regio‐and stereospecific synthesis of oridonin D‐ring aziridinated analogues for the treatment of triple‐negative breast cancer via mediated irreversible covalent warheads. Journal of Medicinal Chemistry, 61(7), 2737–2752. 29528645 10.1021/acs.jmedchem.7b01514 PMC6388690

[fsn33986-bib-0026] Dong, Y. , Zhang, T. , Li, J. , Deng, H. , Song, Y. , Zhai, D. , Peng, Y. , Lu, X. , Liu, M. , Zhao, Y. , & Yi, Z. (2014). Oridonin inhibits tumor growth and metastasis through anti‐angiogenesis by blocking the notch signaling. PLoS One, 9(12), e113830. 25485753 10.1371/journal.pone.0113830 PMC4259472

[fsn33986-bib-0027] Du, Y. , Villeneuve, N. F. , Wang, X. J. , Sun, Z. , Chen, W. , Li, J. , Lou, H. , Wong, P. K. , & Zhang, D. D. (2008). Oridonin confers protection against arsenic‐induced toxicity through activation of the Nrf2‐mediated defensive response. Environmental Health Perspectives, 116(9), 1154–1161. 10.1289/ehp.11464 18795156 PMC2535615

[fsn33986-bib-0028] Duan, H. Q. , Li, M. Y. , Gao, L. , Zhang, J. F. , Wang, W. , Li, Y. , Ma, Y. G. , & Wang, C. B. (2014). Mechanism concerning antitumor effect of oridonin on multiple myeloma cell line U266. Zhongguo Shi Yan Xue Ye Xue Za Zhi, 22(2), 364–369. 24763006 10.7534/j.issn.1009-2137.2014.02.018

[fsn33986-bib-0029] Feng, F. F. , Zhang, D. R. , Tian, K. L. , Lou, H. Y. , Qi, X. L. , Wang, Y. C. , Duan, C. X. , Jia, L. J. , Wang, F. H. , Liu, Y. , & Zhang, Q. (2011). Growth inhibition and induction of apoptosis in MCF‐7 breast cancer cells by oridonin nanosuspension. Drug Delivery, 18(4), 265–271. 21091387 10.3109/10717544.2010.536271

[fsn33986-bib-0030] Feng, N. , Wu, P. , Li, Q. , Mei, Y. , Shi, S. , Yu, J. , Xu, J. , Liu, Y. , & Wang, Y. (2008). Oridonin‐loaded poly (epsilon‐caprolactone)‐poly (ethylene oxide)‐poly (epsilon‐caprolactone) copolymer nanoparticles: Preparation, characterization, and antitumor activity on mice with transplanted hepatoma. Journal of Drug Targeting, 16(6), 479–485. 18604660 10.1080/10611860802200938

[fsn33986-bib-0031] Fujita, E. , Nagao, Y. , Node, M. , Kaneko, K. , Nakazawa, S. , & Kuroda, H. (1976). Antitumor activity of the Isodon diterpenoids: Structural requirements for the activity. Experientia, 32(2), 203–206. 1269612 10.1007/BF01937766

[fsn33986-bib-0032] Gao, F. , Tang, Q. , Yang, P. , Fang, Y. , Li, W. , & Wu, Y. (2010). Apoptosis inducing and differentiation enhancement effect of oridonin on the all‐trans‐retinoic acid‐sensitive and‐resistant acute promyelocytic leukemia cells. International Journal of Laboratory Hematology, 32(1p1), e114–e122. 19302235 10.1111/j.1751-553X.2009.01147.x

[fsn33986-bib-0033] Gao, F. H. , Hu, X. H. , Li, W. , Liu, H. , Zhang, Y. J. , Guo, Z. Y. , Xu, M. H. , Wang, S. T. , Jiang, B. , Liu, F. , & Zhao, Y. Z. (2010). Oridonin induces apoptosis and senescence in colorectal cancer cells by increasing histone hyperacetylation and regulation of p16, p21, p27 and c‐myc. BMC Cancer, 10(1), 1–11. 21054888 10.1186/1471-2407-10-610 PMC2992521

[fsn33986-bib-0034] Gao, F. H. , Liu, F. , Wei, W. E. I. , Liu, L. B. , Xu, M. H. , Guo, Z. Y. , Li, W. , Jiang, B. , & Wu, Y. L. (2012). Oridonin induces apoptosis and senescence by increasing hydrogen peroxide and glutathione depletion in colorectal cancer cells. International Journal of Molecular Medicine, 29(4), 649–655. 22294162 10.3892/ijmm.2012.895 PMC3577350

[fsn33986-bib-0035] Gao, L. , Zhang, D. , Chen, M. , Zheng, T. , & Wang, S. (2007). Preparation and characterization of an oridonin nanosuspension for solubility and dissolution velocity enhancement. Drug Development and Industrial Pharmacy, 33(12), 1332–1339. 18097807 10.1080/03639040701741810

[fsn33986-bib-0036] Gao, S. , Gang, J. , Yu, M. , Xin, G. , & Tan, H. (2021). Computational analysis for identification of early diagnostic biomarkers and prognostic biomarkers of liver cancer based on GEO and TCGA databases and studies on pathways and biological functions affecting the survival time of liver cancer. BMC Cancer, 21(1), 1–15. 34238253 10.1186/s12885-021-08520-1 PMC8268589

[fsn33986-bib-0037] Gao, S. , Tan, H. , & Li, D. (2023). Oridonin suppresses gastric cancer SGC‐7901 cell proliferation by targeting the TNF‐alpha/androgen receptor/TGF‐beta signalling pathway axis. Journal of Cellular and Molecular Medicine, 27(18), 2661–2674. 10.1111/jcmm.17841 37431884 PMC10494293

[fsn33986-bib-0038] Gao, S.‐Y. , Li, J. , Qu, X.‐Y. , Zhu, N. , & Ji, Y.‐B. (2014). Downregulation of Cdk1 and cyclinB1 expression contributes to oridonin‐induced cell cycle arrest at G 2/M phase and growth inhibition in SGC‐7901 gastric cancer cells. Asian Pacific Journal of Cancer Prevention, 15(15), 6437–6441. 25124639 10.7314/apjcp.2014.15.15.6437

[fsn33986-bib-0039] Gao, Z. G. , Ye, Q. X. , & Zhang, T. M. (1993). Synergistic effect of oridonin and cisplatin on cytotoxicity and DNA cross‐link against mouse sarcoma S180 cells in culture. Acta Pharmacologica Sinica, 14(6), 561–564. 8010060

[fsn33986-bib-0040] Gu, Z. , Wang, X. , Qi, R. , Wei, L. , Huo, Y. , Ma, Y. , Shi, L. , Chang, Y. , Li, G. , & Zhou, L. (2015). Oridonin induces apoptosis in uveal melanoma cells by upregulation of Bim and downregulation of fatty acid synthase. Biochemical and Biophysical Research Communications, 457(2), 187–193. 10.1016/j.bbrc.2014.12.086 25545058

[fsn33986-bib-0041] Gui, Y. , Cheng, J. , & Chen, Z. (2021). Oridonin improves the therapeutic effect of lentinan on lung cancer. Experimental and Therapeutic Medicine, 22(2), 1–9. 10.3892/etm.2021.10318 PMC8237276 34194564

[fsn33986-bib-0042] Gui, Z. , Li, S. , Liu, X. , Xu, B. , & Xu, J. (2015). Oridonin alters the expression profiles of microRNAs in BxPC‐3 human pancreatic cancer cells. BMC Complementary and Alternative Medicine, 15(1), 1–10. 25880988 10.1186/s12906-015-0640-5 PMC4399397

[fsn33986-bib-0043] Guo, Y. , Shan, Q. , Gong, Y. , Lin, J. , Yang, X. , & Zhou, R. (2012a). Oridonin in combination with imatinib exerts synergetic anti‐leukemia effect in Ph+ acute lymphoblastic leukemia cells *in vitro* by inhibiting activation of LYN/mTOR signaling pathway. Cancer Biology & Therapy, 13(13), 1244–1254. 22895079 10.4161/cbt.21460 PMC3493431

[fsn33986-bib-0044] Guo, Y. , Shan, Q. Q. , Gong, Y. P. , Lin, J. , Yang, X. , & Zhou, R. Q. (2012b). Anti‐leukemia effect of oridonin on Ph (+) acute lymphoblastic leukemia cell SUP‐B15. Zhonghua Xue Ye Xue Za Zhi = Zhonghua Xueyexue Zazhi, 33(6), 439–443. 22967375

[fsn33986-bib-0045] Han, J. M. , Hong, K. O. , Yang, I. H. , Ahn, C. H. , Jin, B. , Lee, W. , Jung, Y. C. , Kim, K. A. , Shin, J. , Cho, S. D. , & Hong, S. D. (2020). Oridonin induces the apoptosis of mucoepidermoid carcinoma cell lines in a myeloid cell leukemia‐1‐dependent manner. International Journal of Oncology, 57(1), 377–385. 32467983 10.3892/ijo.2020.5061

[fsn33986-bib-0046] Hassannia, B. , Vandenabeele, P. , & Berghe, T. V. (2019). Targeting ferroptosis to iron out cancer. Cancer Cell, 35(6), 830–849. 31105042 10.1016/j.ccell.2019.04.002

[fsn33986-bib-0047] He, H. , Jiang, H. , Chen, Y. , Ye, J. , Wang, A. , Wang, C. , Liu, Q. , Liang, G. , Deng, X. , Jiang, W. , & Zhou, R. (2018). Oridonin is a covalent NLRP3 inhibitor with strong anti‐inflammasome activity. Nature Communications, 9(1), 2550. 10.1038/s41467-018-04947-6 PMC6026158 29959312

[fsn33986-bib-0048] He, S. , Tian, S. , He, X. , Le, X. , Ning, Y. , Chen, J. , Chen, H. , Mu, J. , Xu, K. , Xiang, Q. , & Wu, Y. (2021). Multiple targeted self‐emulsifying compound RGO reveals obvious anti‐tumor potential in hepatocellular carcinoma. Molecular Therapy – Oncolytics, 22, 604–616. 34589579 10.1016/j.omto.2021.08.008 PMC8449031

[fsn33986-bib-0049] He, X. J. , Wang, H. J. , Xia, Y. J. , Ye, Z. Y. , & Tao, H. Q. (2009). Empirical study of oridonin‐induced gastric cancer cells MKN45 apoptosis. Chinese Journal of Gastrointestinal Surgery, 12(6), 607–610. 19921575

[fsn33986-bib-0050] Hu, H. Z. , Yang, Y. B. , Xu, X. D. , Shen, H. W. , Shu, Y. M. , Ren, Z. , Li, X. M. , Shen, H. M. , & Zeng, H. T. (2007). Oridonin induces apoptosis via PI3K/Akt pathway in cervical carcinoma HeLa cell line. Acta Pharmacologica Sinica, 28(11), 1819–1826. 10.1111/j.1745-7254.2007.00667.x 17959034

[fsn33986-bib-0051] Hu, X. , Bai, Z. , Qiao, J. , Li, H. , Xu, S. , Wang, X. , Xu, Y. , Xu, J. , Hua, H. , & Li, D. (2019). Effective enmein‐type mimics of clinical candidate HAO472: Design, synthesis and biological evaluation. European Journal of Medicinal Chemistry, 171, 169–179. 30921757 10.1016/j.ejmech.2019.03.046

[fsn33986-bib-0052] Hu, X. , Wang, Y. , Gao, X. , Xu, S. , Zang, L. , Xiao, Y. , Li, Z. , Hua, H. , Xu, J. , & Li, D. (2020). Recent Progress of Oridonin and its derivatives for the treatment of acute Myelogenous leukemia. Mini Reviews in Medicinal Chemistry, 20(6), 483–497. 10.2174/1389557519666191029121809 31660811

[fsn33986-bib-0053] Huang, H. , Weng, H. , Zhou, H. , & Qu, L. (2014). Attacking c‐Myc: Targeted and combined therapies for cancer. Current Pharmaceutical Design, 20(42), 6543–6554. 25341931 10.2174/1381612820666140826153203

[fsn33986-bib-0054] Huang, H. L. , Weng, H. Y. , Wang, L. Q. , Yu, C. H. , Huang, Q. J. , Zhao, P. P. , Wen, J. Z. , Zhou, H. , & Qu, L. H. (2012). Triggering Fbw7‐mediated proteasomal degradation of c‐Myc by oridonin induces cell growth inhibition and apoptosis. Molecular Cancer Therapeutics, 11(5), 1155–1165. 22389469 10.1158/1535-7163.MCT-12-0066

[fsn33986-bib-0055] Huang, J. , Wu, L. , Tashiro, S.‐I. , Onodera, S. , & Ikejima, T. (2005). A comparison of the signal pathways between the TNF alpha‐and oridonin‐induced murine L929 fibrosarcoma cell death. Acta Medica Okayama, 59(6), 261–270. 16418769 10.18926/AMO/31960

[fsn33986-bib-0056] Hwang, T.‐L. , & Chang, C.‐H. (2023). Oridonin enhances cytotoxic activity of natural killer cells against lung cancer. International Immunopharmacology, 122, 110669. 37480753 10.1016/j.intimp.2023.110669

[fsn33986-bib-0057] Ikezoe, T. , Chen, S. S. , Tong, X.‐J. , Heber, D. , Taguchi, H. , & Koeffler, H. P. (2003). Oridonin induces growth inhibition and apoptosis of a variety of human cancer cells. International Journal of Oncology, 23(4), 1187–1193. 12964003

[fsn33986-bib-0058] Jeon, M. Y. , Seo, S. U. , Woo, S. M. , Min, K. J. , Byun, H. S. , Hur, G. M. , Kang, S. C. , & Kwon, T. K. (2019). Oridonin enhances TRAIL‐induced apoptosis through GALNT14‐mediated DR5 glycosylation. Biochimie, 165, 108–114. 10.1016/j.biochi.2019.07.015 31336136

[fsn33986-bib-0059] Jiang, J. H. , Pi, J. , Jin, H. , & Cai, J. Y. (2019). Oridonin‐induced mitochondria‐dependent apoptosis in esophageal cancer cells by inhibiting PI3K/AKT/mTOR and Ras/Raf pathways. Journal of Cellular Biochemistry, 120(3), 3736–3746. 10.1002/jcb.27654 30229997

[fsn33986-bib-0060] Jilani, K. , Qadri, S. M. , Zelenak, C. , & Lang, F. (2011). Stimulation of suicidal erythrocyte death by oridonin. Archives of Biochemistry and Biophysics, 511(1–2), 14–20. 21575590 10.1016/j.abb.2011.05.001

[fsn33986-bib-0061] Jin, H. , Tan, X. , Liu, X. , & Ding, Y. (2011). Downregulation of AP‐1 gene expression is an initial event in the oridonin‐mediated inhibition of colorectal cancer: Studies *in vitro* and *in vivo* . Journal of Gastroenterology and Hepatology, 26(4), 706–715. 21418301 10.1111/j.1440-1746.2010.06500.x

[fsn33986-bib-0062] Jin, S. , Shen, J.‐N. , Wang, J. , Huang, G. , & Zhou, J.‐G. (2007). Oridonin induced apoptosis through Akt and MAPKs signaling pathways in human osteosarcoma cells. Cancer Biology & Therapy, 6(2), 261–268. 17218775 10.4161/cbt.6.2.3621

[fsn33986-bib-0063] Kang, N. , Cao, S. J. , Zhou, Y. , He, H. , Tashiro, S. I. , Onodera, S. , Qiu, F. , & Ikejima, T. (2015). Inhibition of caspase‐9 by oridonin, a diterpenoid isolated from *Rabdosia rubescens*, augments apoptosis in human laryngeal cancer cells. International Journal of Oncology, 47(6), 2045–2056. 26648189 10.3892/ijo.2015.3186 PMC4665153

[fsn33986-bib-0064] Kang, N. , Zhang, J.‐H. , Qiu, F. , Chen, S. , Tashiro, S.‐I. , Onodera, S. , & Ikejima, T. (2010). Induction of G2/M phase arrest and apoptosis by oridonin in human laryngeal carcinoma cells. Journal of Natural Products, 73(6), 1058–1063. 20496901 10.1021/np9008199

[fsn33986-bib-0065] Kang, N. , Zhang, J.‐H. , Qiu, F. , Tashiro, S.‐I. , Onodera, S. , & Ikejima, T. (2010). Inhibition of EGFR signaling augments oridonin‐induced apoptosis in human laryngeal cancer cells via enhancing oxidative stress coincident with activation of both the intrinsic and extrinsic apoptotic pathways. Cancer Letters, 294(2), 147–158. 20202741 10.1016/j.canlet.2010.01.032

[fsn33986-bib-0066] Khan, K. , Quispe, C. , Javed, Z. , Iqbal, M. J. , Sadia, H. , Raza, S. , Irshad, A. , Salehi, B. , Reiner, Ž. , & Sharifi‐Rad, J. (2020). Resveratrol, curcumin, paclitaxel and miRNAs mediated regulation of PI3K/Akt/mTOR pathway: Go four better to treat bladder cancer. Cancer Cell International, 20, 1–19. 33292283 10.1186/s12935-020-01660-7 PMC7685642

[fsn33986-bib-0067] Khan, K. , Safi, S. , Abbas, A. , Badshah, Y. , Dilshad, E. , Rafiq, M. , Zahra, K. , & Shabbir, M. (2020). Unravelling structure, localization, and genetic crosstalk of KLF3 in human breast cancer. BioMed Research International, 2020, 1–15. 10.1155/2020/1354381 PMC7803292 33490232

[fsn33986-bib-0068] Khan, K. , Shah, H. , Rehman, A. , Badshah, Y. , Ashraf, N. M. , & Shabbir, M. (2022). Influence of PRKCE non‐synonymous variants on protein dynamics and functionality. Human Molecular Genetics, 31(13), 2236–2261. 35137073 10.1093/hmg/ddac029

[fsn33986-bib-0069] Kocarnik, J. M. , Compton, K. , Dean, F. E. , Fu, W. , Gaw, B. L. , Harvey, J. D. , Henrikson, H. J. , Lu, D. , Pennini, A. , Xu, R. , & Ababneh, E. (2022). Cancer incidence, mortality, years of life lost, years lived with disability, and disability‐adjusted life years for 29 cancer groups from 2010 to 2019: A systematic analysis for the global burden of disease study 2019. JAMA Oncology, 8(3), 420–444. 34967848 10.1001/jamaoncol.2021.6987 PMC8719276

[fsn33986-bib-0070] Kou, B. , Yang, Y. , Bai, Y. E. , Shi, Y. H. , Gao, R. X. , Yang, F. L. , Zhang, S. Q. , & Liu, W. (2020). Oridonin induces apoptosis of laryngeal carcinoma via endoplasmic reticulum stress. Cancer Management and Research, 12, 8387–8396. 32982432 10.2147/CMAR.S271759 PMC7494016

[fsn33986-bib-0071] Kwan, H.‐Y. , Yang, Z. , Fong, W.‐F. , Hu, Y.‐M. , Yu, Z.‐L. , & Hsiao, W.‐L. W. (2013). The anticancer effect of oridonin is mediated by fatty acid synthase suppression in human colorectal cancer cells. Journal of Gastroenterology, 48, 182–192. 22722903 10.1007/s00535-012-0612-1

[fsn33986-bib-0072] Leng, X. , Huang, H. , Wang, W. , Sai, N. , You, L. , Yin, X. , & Ni, J. (2018). Zirconium‐porphyrin PCN‐222: pH‐responsive controlled anticancer drug Oridonin. Evidence‐based Complementary and Alternative Medicine, 2018, 3249023. 10.1155/2018/3249023 30622595 PMC6304552

[fsn33986-bib-0073] Leung, C.‐H. , Grill, S. P. , Lam, W. , Han, Q.‐B. , Sun, H.‐D. , & Cheng, Y.‐C. (2005). Novel mechanism of inhibition of nuclear factor‐κB DNA‐binding activity by diterpenoids isolated from *Isodon rubescens* . Molecular Pharmacology, 68(2), 286–297. 15872117 10.1124/mol.105.012765

[fsn33986-bib-0074] Li, C. , Wang, Q. , Shen, S. , Wei, X. , & Li, G. (2018). Oridonin inhibits VEGF‐A‐associated angiogenesis and epithelial‐mesenchymal transition of breast cancer *in vitro* and *in vivo* . Oncology Letters, 16(2), 2289–2298. 30008931 10.3892/ol.2018.8943 PMC6036431

[fsn33986-bib-0075] Li, C. , Zhang, D. , Guo, Y. , Guo, H. , Li, T. , Hao, L. , Zheng, D. , Liu, G. , & Zhang, Q. (2014). Galactosylated bovine serum albumin nanoparticles for parenteral delivery of oridonin: Tissue distribution and pharmacokinetic studies. Journal of Microencapsulation, 31(6), 573–578. 24697186 10.3109/02652048.2014.898705

[fsn33986-bib-0076] Li, D. , Han, T. , Liao, J. , Hu, X. , Xu, S. , Tian, K. , Gu, X. , Cheng, K. , Li, Z. , Hua, H. , & Xu, J. (2016). Oridonin, a promising ent‐kaurane diterpenoid lead compound. International Journal of Molecular Sciences, 17(9), 1395. 27563888 10.3390/ijms17091395 PMC5037675

[fsn33986-bib-0077] Li, D. , Han, T. , Tian, K. , Tang, S. , Xu, S. , Hu, X. , Wang, L. , Li, Z. , Hua, H. , & Xu, J. (2016). Novel nitric oxide‐releasing spirolactone‐type diterpenoid derivatives with *in vitro* synergistic anticancer activity as apoptosis inducer. Bioorganic & Medicinal Chemistry Letters, 26(17), 4191–4196. 27491707 10.1016/j.bmcl.2016.07.059

[fsn33986-bib-0078] Li, D. , Wang, H. , Ding, Y. , Zhang, Z. , Zheng, Z. , Dong, J. , Kim, H. , Meng, X. , Zhou, Q. , Zhou, J. , & Fang, L. (2018). Targeting the NRF‐2/RHOA/ROCK signaling pathway with a novel aziridonin, YD0514, to suppress breast cancer progression and lung metastasis. Cancer Letters, 424, 97–108. 29580806 10.1016/j.canlet.2018.03.029

[fsn33986-bib-0079] Li, D. , Wang, L. , Cai, H. , Zhang, Y. , & Xu, J. (2012). Synthesis and biological evaluation of novel furozan‐based nitric oxide‐releasing derivatives of oridonin as potential anti‐tumor agents. Molecules, 17(6), 7556–7568. 22710829 10.3390/molecules17067556 PMC6268409

[fsn33986-bib-0080] Li, D. , Wu, L.‐J. , Tashiro, S.‐I. , Onodera, S. , & Ikejima, T. (2007a). Oridonin‐induced A431 cell apoptosis partially through blockage of the Ras/Raf/ERK signal pathway. Journal of Pharmacological Sciences, 103(1), 56–66. 17251686 10.1254/jphs.fpj06016x

[fsn33986-bib-0081] Li, D. , Wu, L.‐J. , Tashiro, S.‐I. , Onodera, S. , & Ikejima, T. (2007b). Oridonin inhibited the tyrosine kinase activity and induced apoptosis in human epidermoid carcinoma A431 cells. Biological and Pharmaceutical Bulletin, 30(2), 254–260. 17268061 10.1248/bpb.30.254

[fsn33986-bib-0082] Li, D. , Xu, S. , Cai, H. , Pei, L. , Wang, L. , Wu, X. , Yao, H. , Jiang, J. , Sun, Y. , & Xu, J. (2013). Library construction and biological evaluation of Enmein‐type Diterpenoid analogues as potential anticancer agents. ChemMedChem, 8(5), 812–818. 23520191 10.1002/cmdc.201200559

[fsn33986-bib-0083] Li, D. A. N. , Wu, L.‐J. , Tashiro, S.‐I. , Onodera, S. , & Ikejima, T. (2008). Oridonin induces human epidermoid carcinoma A431 cell apoptosis through tyrosine kinase and mitochondrial pathway. Journal of Asian Natural Products Research, 10(1), 77–87. 18058384 10.1080/10286020701273866

[fsn33986-bib-0084] Li, F. F. , Yi, S. , Wen, L. , He, J. , Yang, L. J. , Zhao, J. , Zhang, B. P. , Cui, G. H. , & Chen, Y. (2014). Oridonin induces NPM mutant protein translocation and apoptosis in NPM1c+ acute myeloid leukemia cells *in vitro* . Acta Pharmacologica Sinica, 35(6), 806–813. 24902788 10.1038/aps.2014.25 PMC4086383

[fsn33986-bib-0085] Li, H. , Mu, J. , Sun, J. , Xu, S. , Liu, W. , Xu, F. , Li, Z. , Xu, J. , Hua, H. , & Li, D. (2020). Hydrogen sulfide releasing oridonin derivatives induce apoptosis through extrinsic and intrinsic pathways. European Journal of Medicinal Chemistry, 187, 111978. 31877536 10.1016/j.ejmech.2019.111978

[fsn33986-bib-0086] Li, J. , Wu, Y. , Wang, D. , Zou, L. , Fu, C. , Zhang, J. , & Leung, G. P. (2019). Oridonin synergistically enhances the anti‐tumor efficacy of doxorubicin against aggressive breast cancer via pro‐apoptotic and anti‐angiogenic effects. Pharmacological Research, 146, 104313. 10.1016/j.phrs.2019.104313 31202781

[fsn33986-bib-0087] Li, W. , & Ma, L. (2019). Synergistic antitumor activity of oridonin and valproic acid on HL‐60 leukemia cells. Journal of Cellular Biochemistry, 120(4), 5620–5627. 10.1002/jcb.27845 30320906

[fsn33986-bib-0088] Li, X. , Chen, W. , Liu, K. , Zhang, S. , Yang, R. , Liu, K. , Li, D. , & Huang, Y. (2020). Oridonin sensitizes hepatocellular carcinoma to the anticancer effect of Sorafenib by targeting the Akt pathway. Cancer Management and Research, 12, 8081–8091. 10.2147/cmar.S257482 32982405 PMC7494228

[fsn33986-bib-0089] Li, X. , Wang, J. , Ye, Z. , & Li, J.‐C. (2012). Oridonin up‐regulates expression of P21 and induces autophagy and apoptosis in human prostate cancer cells. International Journal of Biological Sciences, 8(6), 901–912. 22745580 10.7150/ijbs.4554 PMC3385012

[fsn33986-bib-0090] Li, X. , Zhang, C.‐T. , Ma, W. , Xie, X. , & Huang, Q. (2021). Oridonin: A review of its pharmacology, pharmacokinetics and toxicity. Frontiers in Pharmacology, 12, 645824. 34295243 10.3389/fphar.2021.645824 PMC8289702

[fsn33986-bib-0091] Li, Y. , Wang, Y. , Wang, S. , Gao, Y. , Zhang, X. , & Lu, C. (2015). Oridonin phosphate‐induced autophagy effectively enhances cell apoptosis of human breast cancer cells. Medical Oncology, 32, 1–8. 10.1007/s12032-014-0365-1 25491140

[fsn33986-bib-0092] Lin, T.‐Y. , Lee, C.‐C. , Chen, K.‐C. , Lin, C.‐J. , & Shih, C.‐M. (2015). Inhibition of RNA transportation induces glioma cell apoptosis via downregulation of RanGAP1 expression. Chemico‐Biological Interactions, 232, 49–57. 25746355 10.1016/j.cbi.2015.02.019

[fsn33986-bib-0093] List, P. (2013). The Plant List. Version 1.1. *Published on the Internet* .

[fsn33986-bib-0094] Liu, D.‐L. , Bu, H.‐Q. , Jin, H.‐M. , Zhao, J.‐F. , Li, Y. , & Huang, H. (2014). Enhancement of the effects of gemcitabine against pancreatic cancer by oridonin via the mitochondrial caspase‐dependent signaling pathway. Molecular Medicine Reports, 10(6), 3027–3034. 25242370 10.3892/mmr.2014.2584

[fsn33986-bib-0095] Liu, H. , Qian, C. , & Shen, Z. (2014). Anti‐tumor activity of oridonin on SNU‐5 subcutaneous xenograft model via regulation of c‐met pathway. Tumor Biology, 35, 9139–9146. 24916572 10.1007/s13277-014-2178-4

[fsn33986-bib-0096] Liu, J. , Huang, R. , Lin, D. , Wu, X. , Peng, J. , Lin, Q. , Pan, X. , Zhang, M. , Hou, M. , & Chen, F. (2005). Apoptotic effect of oridonin on NB4 cells and its mechanism. Leukemia & Lymphoma, 46(4), 593–597. 16019488 10.1080/10428190400019800

[fsn33986-bib-0097] Liu, J. J. , Huang, R. W. , Lin, D. J. , Peng, J. , Wu, X. Y. , Pan, X. L. , Li, M. Q. , & Lin, Q. (2004). Anti‐proliferative effects of oridonin on SPC‐A‐1 cells and its mechanism of action. Journal of International Medical Research, 32(6), 617–625. 15587755 10.1177/147323000403200606

[fsn33986-bib-0098] Liu, J. J. , Huang, R. W. , Lin, D. J. , Wu, X. Y. , Peng, J. , Pan, X. L. , Lin, Q. , Hou, M. , Zhang, M. H. & Chen, F. (2006). Antiproliferation effects of oridonin on HPB‐ALL cells and its mechanisms of action. American Journal of Hematology, 81(2), 86–94. 16432862 10.1002/ajh.20524

[fsn33986-bib-0099] Liu, Q. Q. , Wang, H. L. , Chen, K. , Wang, S. B. , Xu, Y. , Ye, Q. , & Sun, Y. W. (2016). Oridonin derivative ameliorates experimental colitis by inhibiting activated T‐cells and translocation of nuclear factor‐kappa B. Journal of Digestive Diseases, 17(2), 104–112. 26718746 10.1111/1751-2980.12314

[fsn33986-bib-0100] Liu, W. , Wang, X. , Wang, L. , Mei, Y. , Yun, Y. , Yao, X. , Chen, Q. , Zhou, J. , & Kou, B. (2022). Oridonin represses epithelial‐mesenchymal transition and angiogenesis of thyroid cancer via downregulating JAK2/STAT3 signaling. International Journal of Medical Sciences, 19(6), 965–974. 35813296 10.7150/ijms.70733 PMC9254367

[fsn33986-bib-0101] Liu, W. , Zhao, B. , Li, Y. C. , & Liu, H. M. (2011). NMR spectra and structures of oridonin derivatives complexes with β‐cyclodextrin. Magnetic Resonance in Chemistry, 49(9), 611–615. 21815208 10.1002/mrc.2770

[fsn33986-bib-0102] Liu, X. , Xu, J. , Zhou, J. , & Shen, Q. (2021). Oridonin and its derivatives for cancer treatment and overcoming therapeutic resistance. Genes & Diseases, 8(4), 448–462. 34179309 10.1016/j.gendis.2020.06.010 PMC8209342

[fsn33986-bib-0103] Liu, Y. , Liu, J.‐H. , Chai, K. , Tashiro, S.‐I. , Onodera, S. , & Ikejima, T. (2013). Inhibition of c‐met promoted apoptosis, autophagy and loss of the mitochondrial transmembrane potential in oridonin‐induced A549 lung cancer cells. Journal of Pharmacy and Pharmacology, 65(11), 1622–1642. 24102522 10.1111/jphp.12140

[fsn33986-bib-0104] Liu, Y. , Liu, Y. Z. , Zhang, R. X. , Wang, X. , Meng, Z. J. , Huang, J. , Wu, K. , Luo, J. Y. , Zuo, G. W. , Chen, L. , & Yin, L. J. (2014). Oridonin inhibits the proliferation of human osteosarcoma cells by suppressing Wnt/β‐catenin signaling. International Journal of Oncology, 45(2), 795–803. 24859848 10.3892/ijo.2014.2456

[fsn33986-bib-0105] Liu, Y. , Shi, Q.‐F. , Qi, M. , Tashiro, S.‐I. , Onodera, S. , & Ikejima, T. (2012). Interruption of hepatocyte growth factor signaling augmented oridonin‐induced death in human non‐small cell lung cancer A549 cells via c‐met‐nuclear factor‐κB‐cyclooxygenase‐2 and c‐met‐Bcl‐2‐caspase‐3 pathways. Biological and Pharmaceutical Bulletin, 35(7), 1150–1158. 22791165 10.1248/bpb.b12-00197

[fsn33986-bib-0106] Liu, Y.‐Q. , You, S. , Zhang, C.‐L. , Tashiro, S.‐I. , Onodera, S. , & Ikejima, T. (2005). Oridonin enhances phagocytosis of UV‐irradiated apoptotic U937 cells. Biological and Pharmaceutical Bulletin, 28(3), 461–467. 15744069 10.1248/bpb.28.461

[fsn33986-bib-0107] Lou, H. , Gao, L. , Wei, X. , Zhang, Z. , Zheng, D. , Zhang, D. , Zhang, X. , Li, Y. , & Zhang, Q. (2011). Oridonin nanosuspension enhances anti‐tumor efficacy in SMMC‐7721 cells and H22 tumor bearing mice. Colloids and Surfaces B: Biointerfaces, 87(2), 319–325. 21676599 10.1016/j.colsurfb.2011.05.037

[fsn33986-bib-0108] Lu, J. , Chen, X. , Qu, S. , Yao, B. , Xu, Y. , Wu, J. , Jin, Y. , & Ma, C. (2017). Oridonin induces G(2)/M cell cycle arrest and apoptosis via the PI3K/Akt signaling pathway in hormone‐independent prostate cancer cells. Oncology Letters, 13(4), 2838–2846. 10.3892/ol.2017.5751 28454475 PMC5403405

[fsn33986-bib-0109] Ma, Y. C. , Ke, Y. , Zi, X. , Zhao, F. , Yuan, L. , Zhu, Y. L. , Fan, X. X. , Zhao, N. M. , Li, Q. Y. , Qin, Y. H. , & Liu, H. M. (2016). Induction of the mitochondria‐mediated apoptosis in human esophageal cancer cells by DS2, a newly synthetic diterpenoid analog, is regulated by Bax and caused by generation of reactive oxygen species. Oncotarget, 7(52), 86211–86224. 27863415 10.18632/oncotarget.13367 PMC5349908

[fsn33986-bib-0110] Malik, S. K. , Ahmed, M. , & Khan, F. (2018). Identification of novel anticancer terpenoids from Prosopis juliflora (Sw) DC (Leguminosae) pods. Tropical Journal of Pharmaceutical Research, 17(4), 661–668.

[fsn33986-bib-0111] Marks, L. S. , DiPaola, R. S. , Nelson, P. , Chen, S. , Heber, D. , Belldegrun, A. S. , Lowe, F. C. , Fan, J. , Leaders, F. E., Jr. , Pantuck, A. J. , & Tyler, V. E. (2002). PC‐SPES: Herbal formulation for prostate cancer. Urology, 60(3), 369–375. 12350462 10.1016/s0090-4295(02)01913-1

[fsn33986-bib-0112] Meade‐Tollin, L. C. , Wijeratne, E. K. , Cooper, D. , Guild, M. , Jon, E. , Fritz, A. , Zhou, G. X. , Whitesell, L. , Liang, J. Y. , & Gunatilaka, A. L. (2004). Ponicidin and oridonin are responsible for the antiangiogenic activity of *Rabdosia rubescens*, a constituent of the herbal supplement PC SPES. Journal of Natural Products, 67(1), 2–4. 14738375 10.1021/np0304114

[fsn33986-bib-0113] Ming, M. , Sun, F.‐Y. , Zhang, W.‐T. , & Liu, J.‐K. (2016). Therapeutic effect of oridonin on mice with prostate cancer. Asian Pacific Journal of Tropical Medicine, 9(2), 184–187. 26919953 10.1016/j.apjtm.2016.01.007

[fsn33986-bib-0114] Node, M. , Sai, M. , Fuji, K. , Fujita, E. , Takeda, S. , & Unemi, N. (1983). Antitumor activity of diterpenoids, trichorabdals A, B, and C, and the related compounds: Synergism of two active sites. Chemical and Pharmaceutical Bulletin, 31(4), 1433–1436. 6627520 10.1248/cpb.31.1433

[fsn33986-bib-0115] Owona, B. A. , & Schluesener, H. J. (2015). Molecular insight in the multifunctional effects of oridonin. Drugs in R&D, 15, 233–244. 26290464 10.1007/s40268-015-0102-z PMC4561052

[fsn33986-bib-0021] Piaz, D. , Fabrizio, R. C. , Lepore, L. , Vassallo, A. , Malafronte, N. , Lauro, G. , Bifulco, G. , Belisario, M. A. , & De Tommasi, N. (2013). Chemical proteomics reveals HSP70 1A as a target for the anticancer diterpene oridonin in Jurkat cells. Journal of Proteomics, 82, 14–26. 23416714 10.1016/j.jprot.2013.01.030

[fsn33986-bib-0116] Piñeros, M. , Mery, L. , Soerjomataram, I. , Bray, F. , & Steliarova‐Foucher, E. (2021). Scaling up the surveillance of childhood cancer: A global roadmap. JNCI Journal of the National Cancer Institute, 113(1), 9–15. 32433739 10.1093/jnci/djaa069 PMC7781445

[fsn33986-bib-0117] Qi, X. , Zhang, D. , Xu, X. , Feng, F. , Ren, G. , Chu, Q. , Zhang, Q. , & Tian, K. (2012). Oridonin nanosuspension was more effective than free oridonin on G2/M cell cycle arrest and apoptosis in the human pancreatic cancer PANC‐1 cell line. International Journal of Nanomedicine, 7(52), 1793–1804. 22619528 10.2147/IJN.S29483 PMC3356194

[fsn33986-bib-0118] Ren, D. L. , Ghoorun, R. A. , Wu, X. H. , Chen, H. L. , Zhou, Q. , & Wu, X. B. (2020). Oridonin induces apoptosis in HGC‐27 cells by activating the JNK signaling pathway. Oncology Letters, 19(1), 255–260. 10.3892/ol.2019.11104 31897137 PMC6923929

[fsn33986-bib-0119] Ren, K. K. , Wang, H. Z. , Xie, L. P. , Chen, D. W. , Liu, X. , Sun, J. , Nie, Y. C. , & Zhang, R. Q. (2006). The effects of oridonin on cell growth, cell cycle, cell migration and differentiation in melanoma cells. Journal of Ethnopharmacology, 103(2), 176–180. 16169170 10.1016/j.jep.2005.07.020

[fsn33986-bib-0120] Shabbir, M. , Badshah, Y. , Khan, K. , Trembley, J. H. , Rizwan, A. , Faraz, F. , Shah, S. A. , Farooqi, M. , Ashraf, N. M. , Afsar, T. , & Almajwal, A. (2022). Association of CTLA‐4 and IL‐4 polymorphisms in viral induced liver cancer. BMC Cancer, 22(1), 518. 35525950 10.1186/s12885-022-09633-x PMC9080112

[fsn33986-bib-0121] Shabbir, M. , Mukhtar, H. , Syed, D. , Razak, S. , Afsar, T. , Almajwal, A. , Badshah, Y. , & Aldisi, D. (2021). Tissue microarray profiling and integrative proteomics indicate the modulatory potential of *Maytenus royleanus* in inhibition of overexpressed TPD52 in prostate cancers. Scientific Reports, 11(1), 1–21. 34099820 10.1038/s41598-021-91408-8 PMC8184821

[fsn33986-bib-0122] Shen, Q.‐K. , Chen, Z.‐A. , Zhang, H.‐J. , Li, J.‐L. , Liu, C.‐F. , Gong, G.‐H. , & Quan, Z.‐S. (2018). Design and synthesis of novel oridonin analogues as potent anticancer agents. Journal of Enzyme Inhibition and Medicinal Chemistry, 33(1), 324–333. 29303372 10.1080/14756366.2017.1419219 PMC6054517

[fsn33986-bib-0123] Shen, Q.‐K. , Deng, H. , Wang, S.‐B. , Tian, Y.‐S. , & Quan, Z.‐S. (2019). Synthesis, and evaluation of *in vitro* and *in vivo* anticancer activity of 14‐substituted oridonin analogs: A novel and potent cell cycle arrest and apoptosis inducer through the p53‐MDM2 pathway. European Journal of Medicinal Chemistry, 173, 15–31. 30981113 10.1016/j.ejmech.2019.04.005

[fsn33986-bib-0124] Shen, Y. , & Ma, H. (2022). Oridonin‐loaded lipid‐coated calcium phosphate nanoparticles: Preparation, characterization, and application in A549 lung cancer. Pharmaceutical Development and Technology, 27(5), 598–605. 35734959 10.1080/10837450.2022.2090958

[fsn33986-bib-0125] Song, M. , Liu, X. , Liu, K. , Zhao, R. , Huang, H. , Shi, Y. , Zhang, M. , Zhou, S. , Xie, H. , Chen, H. , & Li, Y. (2019). Targeting AKT with oridonin inhibits growth of esophageal squamous cell carcinoma *in vitro* and patient‐derived xenografts *in vivo* . Cancer Research, 79(13_Supplement), 4299. 10.1158/1535-7163.MCT-17-0823 PMC6715294 29695636

[fsn33986-bib-0126] Sun, K. W. , Ma, Y. Y. , Guan, T. P. , Xia, Y. J. , Shao, C. M. , Chen, L. G. , Ren, Y. J. , Yao, H. B. , Yang, Q. , & He, X. J. (2012). Oridonin induces apoptosis in gastric cancer through Apaf‐1, cytochrome c and caspase‐3 signaling pathway. World Journal of Gastroenterology: WJG, 18(48), 7166–7174. 23326121 10.3748/wjg.v18.i48.7166 PMC3544018

[fsn33986-bib-0127] Sun, P. , Wu, G. , Qiu, Z. , & Chen, Y. (2014). Preparation of L‐alanine‐(14‐oridonin) ester trifluoroacetate for treatment of cancer. Patent Application CN104017000A, 3.

[fsn33986-bib-0128] Sun, Q. , Xie, L. , Song, J. , & Li, X. (2020). Evodiamine: A review of its pharmacology, toxicity, pharmacokinetics and preparation researches. Journal of Ethnopharmacology, 262, 113164. 32738391 10.1016/j.jep.2020.113164

[fsn33986-bib-0129] Sung, H. , Ferlay, J. , Siegel, R. L. , Laversanne, M. , Soerjomataram, I. , Jemal, A. , & Bray, F. (2021). Global cancer statistics 2020: GLOBOCAN estimates of incidence and mortality worldwide for 36 cancers in 185 countries. CA: A Cancer Journal for Clinicians, 71(3), 209–249. 33538338 10.3322/caac.21660

[fsn33986-bib-0130] Tan, W. , Lu, J. , Huang, M. , Li, Y. , Chen, M. , Wu, G. , Gong, J. , Zhong, Z. , Xu, Z. , Dang, Y. , & Guo, J. (2011). Anti‐cancer natural products isolated from Chinese medicinal herbs. Chinese Medicine, 6(1), 27. 21777476 10.1186/1749-8546-6-27 PMC3149025

[fsn33986-bib-0131] Tian, L. , Sheng, D. , Li, Q. , Guo, C. , & Zhu, G. (2019). Preliminary safety assessment of oridonin in zebrafish. Pharmaceutical Biology, 57(1), 632–640. 31545911 10.1080/13880209.2019.1662457 PMC6764400

[fsn33986-bib-0132] Tiwari, R. V. , Parajuli, P. , & Sylvester, P. W. (2015). Synergistic anticancer effects of combined γ‐tocotrienol and oridonin treatment is associated with the induction of autophagy. Molecular and Cellular Biochemistry, 408, 123–137. 26112904 10.1007/s11010-015-2488-x

[fsn33986-bib-0133] Vasaturo, M. , Cotugno, R. , Fiengo, L. , Vinegoni, C. , Dal Piaz, F. , & De Tommasi, N. (2018). The anti‐tumor diterpene oridonin is a direct inhibitor of Nucleolin in cancer cells. Scientific Reports, 8(1), 16735. 10.1038/s41598-018-35088-x 30425290 PMC6233161

[fsn33986-bib-0134] Wang, C. , Jiang, L. , Wang, S. , Shi, H. , Wang, J. , Wang, R. , Li, Y. , Dou, Y. , Liu, Y. , Hou, G. , & Ke, Y. (2015). The antitumor activity of the novel compound jesridonin on human esophageal carcinoma cells. PLoS One, 10(6), e0130284. 26103161 10.1371/journal.pone.0130284 PMC4477902

[fsn33986-bib-0135] Wang, H. , Wang, Y.‐F. , Liu, T.‐G. , Xiang, X.‐L. , & Huang, S.‐L. (2014). Experimental study on anti‐pancreatic cancer effect of oridonin. Journal of Chinese Medicinal Materials, 37(7), 1230–1233. 25566662

[fsn33986-bib-0136] Wang, H. , Ye, Y. , Chu, J.‐H. , Zhu, G.‐Y. , Fong, W.‐F. , & Yu, Z.‐L. (2011). Proteomic and functional analyses reveal the potential involvement of endoplasmic reticulum stress and α‐CP1 in the anticancer activities of oridonin in HepG2 cells. Integrative Cancer Therapies, 10(2), 160–167. 20926737 10.1177/1534735410383171

[fsn33986-bib-0137] Wang, H. , Ye, Y. , Chu, J.‐H. , Zhu, G.‐Y. , Li, Y.‐W. , Fong, D. W. F. , & Yu, Z.‐L. (2010). Oridonin induces G2/M cell cycle arrest and apoptosis through MAPK and p53 signaling pathways in HepG2 cells. Oncology Reports, 24(3), 647–651. 20664969

[fsn33986-bib-0138] Wang, H. , Ye, Y. , Pan, S.‐Y. , Zhu, G.‐Y. , Li, Y.‐W. , Fong, D. W. F. , & Yu, Z.‐L. (2011). Proteomic identification of proteins involved in the anticancer activities of oridonin in HepG2 cells. Phytomedicine, 18(2–3), 163–169. 20724128 10.1016/j.phymed.2010.06.011

[fsn33986-bib-0139] Wang, H. , Ye, Y. , & Yu, Z.‐L. (2014). Proteomic and functional analyses demonstrate the involvement of oxidative stress in the anticancer activities of oridonin in HepG2 cells. Oncology Reports, 31(5), 2165–2172. 24627081 10.3892/or.2014.3081

[fsn33986-bib-0140] Wang, L. , Li, D. , Xu, S. , Cai, H. , Yao, H. , Zhang, Y. , Jiang, J. , & Xu, J. (2012). The conversion of oridonin to spirolactone‐type or enmein‐type diterpenoid: Synthesis and biological evaluation of ent‐6, 7‐seco‐oridonin derivatives as novel potential anticancer agents. European Journal of Medicinal Chemistry, 52, 242–250. 22483090 10.1016/j.ejmech.2012.03.024

[fsn33986-bib-0141] Wang, S. , Yang, H. , Yu, L. , Jin, J. , Qian, L. , Zhao, H. , Xu, Y. , & Zhu, X. (2014). Oridonin attenuates Aβ1‐42‐induced neuroinflammation and inhibits NF‐κB pathway. PLoS One, 9(8), e104745. 10.1371/journal.pone.0104745 25121593 PMC4133239

[fsn33986-bib-0142] Wang, S. , Zhong, Z. , Wan, J. , Tan, W. , Wu, G. , Chen, M. , & Wang, Y. (2013). Oridonin induces apoptosis, inhibits migration and invasion on highly‐metastatic human breast cancer cells. The American Journal of Chinese Medicine, 41(01), 177–196. 23336515 10.1142/S0192415X13500134

[fsn33986-bib-0143] Wang, S. Q. , Wang, C. , Wang, J. W. , Yang, D. X. , Wang, R. , Wang, C. J. , Li, H. J. , Shi, H. G. , Ke, Y. , & Liu, H. M. (2017). Geridonin, a novel derivative of oridonin, inhibits proliferation of MGC 803 cells both *in vitro* and *in vivo* through elevating the intracellular ROS. Journal of Pharmacy and Pharmacology, 69(2), 213–221. 28028809 10.1111/jphp.12678

[fsn33986-bib-0144] Wang, Y. , Lv, H. , Dai, C. , Wang, X. , Yin, Y. , & Chen, Z. (2021). Oridonin dose‐dependently modulates the cell senescence and apoptosis of gastric cancer cells. Evidence‐based Complementary and Alternative Medicine, 2021, 12. 10.1155/2021/5023536 PMC8595004 34795783

[fsn33986-bib-0145] Wang, Y. , & Zhu, Z. (2019). Oridonin inhibits metastasis of human ovarian cancer cells by suppressing the mTOR pathway. Archives of Medical Science, 15(4), 1017–1027. 31360196 10.5114/aoms.2018.77068 PMC6657258

[fsn33986-bib-0146] Wang, Y.‐Y. , Lv, Y.‐F. , Lu, L. , & Cai, L. (2014). Oridonin inhibits mTOR signaling and the growth of lung cancer tumors. Anti‐Cancer Drugs, 25(10), 1192–1200. 25075795 10.1097/CAD.0000000000000154

[fsn33986-bib-0147] Weng, H. , Huang, H. , Dong, B. , Zhao, P. , Zhou, H. , & Qu, L. (2014). Inhibition of miR‐17 and miR‐20a by oridonin triggers apoptosis and reverses chemoresistance by depressing BIM‐SOridonin reverses chemoresistance via miR‐17/20a‐BIM‐S pathway. Cancer Research, 74(16), 4409–4419. 24872388 10.1158/0008-5472.CAN-13-1748

[fsn33986-bib-0148] Xia, S. , Zhang, X. , Li, C. , & Guan, H. (2017). Oridonin inhibits breast cancer growth and metastasis through blocking the notch signaling. Saudi Pharmaceutical Journal, 25(4), 638–643. 28579904 10.1016/j.jsps.2017.04.037 PMC5447451

[fsn33986-bib-0149] Xu, B. , Shen, W. , Liu, X. , Zhang, T. , Ren, J. , Fan, Y. , & Xu, J. (2015). Oridonin inhibits BxPC‐3 cell growth through cell apoptosis. Acta Biochimica et Biophysica Sinica, 47(3), 164–173. 25651847 10.1093/abbs/gmu134

[fsn33986-bib-0150] Xu, J. , Yang, J. , Ran, Q. , Wang, L. , Liu, J. , Wang, Z. , Wu, X. , Hua, W. , Yuan, S. , Zhang, L. , & Shen, M. (2008). Synthesis and biological evaluation of novel 1‐O‐and 14‐O‐derivatives of oridonin as potential anticancer drug candidates. Bioorganic & Medicinal Chemistry Letters, 18(16), 4741–4744. 18644718 10.1016/j.bmcl.2008.06.097

[fsn33986-bib-0151] Xu, J. , Zhao, J. H. , Liu, Y. , Feng, N. P. , & Zhang, Y. T. (2012). RGD‐modified poly(D,L‐lactic acid) nanoparticles enhance tumor targeting of oridonin. International Journal of Nanomedicine, 7, 211–219. 22275836 10.2147/IJN.S27581 PMC3263413

[fsn33986-bib-0152] Xu, L. , Bi, Y. , Xu, Y. , Zhang, Z. , Xu, W. , Zhang, S. , & Chen, J. (2020). Oridonin inhibits the migration and epithelial‐to‐mesenchymal transition of small cell lung cancer cells by suppressing FAK‐ERK1/2 signalling pathway. Journal of Cellular and Molecular Medicine, 24(8), 4480–4493. 10.1111/jcmm.15106 32168416 PMC7176879

[fsn33986-bib-0153] Xu, S. , Pei, L. , Wang, C. , Zhang, Y. K. , Li, D. , Yao, H. , Wu, X. , Chen, Z. S. , Sun, Y. , & Xu, J. (2014). Novel hybrids of natural oridonin‐bearing nitrogen mustards as potential anticancer drug candidates. ACS Medicinal Chemistry Letters, 5(7), 797–802. 25050168 10.1021/ml500141f PMC4094252

[fsn33986-bib-0154] Xu, S. , Yao, H. , Hu, M. , Li, D. , Zhu, Z. , Xie, W. , Yao, H. , Wu, L. , Chen, Z. S. , & Xu, J. (2017). 6, 7‐Seco‐ent‐kauranoids derived from oridonin as potential anticancer agents. Journal of Natural Products, 80(9), 2391–2398. 28901767 10.1021/acs.jnatprod.7b00057

[fsn33986-bib-0155] Xu, S. , Yao, H. , Luo, S. , Zhang, Y.‐K. , Yang, D.‐H. , Li, D. , Wang, G. , Hu, M. , Qiu, Y. , Wu, X. , Yao, H. , Xie, W. , Chen, Z. S. , & Xu, J. (2017). A novel potent anticancer compound optimized from a natural oridonin scaffold induces apoptosis and cell cycle arrest through the mitochondrial pathway. Journal of Medicinal Chemistry, 60(4), 1449–1468. 28165738 10.1021/acs.jmedchem.6b01652

[fsn33986-bib-0156] Xue, B. , Wang, Y. , Tang, X. , Xie, P. , Luo, F. , Wu, C. , & Qian, Z. (2012). Biodegradable self‐assembled MPEG‐PCL micelles for hydrophobic oridonin delivery *in vitro* . Journal of Biomedical Nanotechnology, 8(1), 80–89. 22515096 10.1166/jbn.2012.1358

[fsn33986-bib-0157] Yan, S.‐L. , Huang, C.‐S. , Mong, M.‐C. , & Yin, M.‐C. (2021). Oridonin attenuates the effects of chronic alcohol consumption inducing oxidative, glycative and inflammatory injury in the mouse liver. In Vivo, 35(4), 2141–2149. 34182490 10.21873/invivo.12484 PMC8286538

[fsn33986-bib-0158] Yan, Y. , Tan, R. Z. , Liu, P. , Li, J. C. , Zhong, X. , Liao, Y. , Lin, X. , Wei, C. , & Wang, L. (2020). Oridonin alleviates IRI‐induced kidney injury by inhibiting inflammatory response of macrophages via AKT‐related pathways. Medical Science Monitor: International Medical Journal of Experimental and Clinical Research, 26, e921114. 32362652 10.12659/MSM.921114 PMC7219002

[fsn33986-bib-0159] Yang, H. , Wang, J. , Khan, S. , Zhang, Y. , Zhu, K. , Zhou, E. , Gong, M. , Liu, B. , Kan, Q. , & Zhang, Q. (2022). Selective synergistic anticancer effects of cisplatin and oridonin against human p53‐mutant esophageal squamous carcinoma cells. Anti‐Cancer Drugs, 33(1), e444. 34520434 10.1097/CAD.0000000000001237 PMC8670348

[fsn33986-bib-0160] Yang, J. , Jiang, H. , Wang, C. , Yang, B. , Zhao, L. , Hu, D. , Qiu, G. , Dong, X. , & Xiao, B. (2015). Oridonin triggers apoptosis in colorectal carcinoma cells and suppression of microRNA‐32 expression augments oridonin‐mediated apoptotic effects. Biomedicine & Pharmacotherapy, 72, 125–134. 26054686 10.1016/j.biopha.2015.04.016

[fsn33986-bib-0161] Yao, H. , Xie, S. , Ma, X. , Liu, J. , Wu, H. , Lin, A. , Yao, H. , Li, D. , Xu, S. , Yang, D. H. , & Chen, Z. S. (2020). Identification of a potent oridonin analogue for treatment of triple‐negative breast cancer. Journal of Medicinal Chemistry, 63(15), 8157–8178. 32610904 10.1021/acs.jmedchem.0c00408

[fsn33986-bib-0162] Yao, Z. , Xie, F. , Li, M. , Liang, Z. , Xu, W. , Yang, J. , Liu, C. , Li, H. , Zhou, H. , & Qu, L. H. (2017). Oridonin induces autophagy via inhibition of glucose metabolism in p53‐mutated colorectal cancer cells. Cell Death & Disease, 8(2), e2633. 10.1038/cddis.2017.35 28230866 PMC5386482

[fsn33986-bib-0163] Ye, L. H. , Chen, Y. L. , Tao, S. X. , Jiang, X. Q. , Li, W. J. , Qian, W. L. , & He, J. S. (2010). Autophagy of prostate cancer PC‐3 cells under the induction of oridonin. Zhonghua Yi Xue Za Zhi, 90(42), 2984–2988. 21211311

[fsn33986-bib-0164] Ye, L. H. , Li, W. J. , Jiang, X. Q. , Chen, Y. L. , Tao, S. X. , Qian, W. L. , & He, J. S. (2012). Study on the autophagy of prostate cancer PC‐3 cells induced by oridonin. The Anatomical Record: Advances in Integrative Anatomy and Evolutionary Biology, 295(3), 417–422. 22190546 10.1002/ar.21528

[fsn33986-bib-0165] Ye, Y. C. , Wang, H. J. , Xu, L. , Liu, W. W. , Liu, B. B. , Tashiro, S. , Onodera, S. , & Ikejima, T. (2012). Oridonin induces apoptosis and autophagy in murine fibrosarcoma L929 cells partly via NO‐ERK‐p53 positive‐feedback loop signaling pathway. Acta Pharmacologica Sinica, 33(8), 1055–1061. 22842735 10.1038/aps.2012.53 PMC4085664

[fsn33986-bib-0166] Yi, S. , Chen, Y. , Wen, L. , Yang, L. , & Cui, G. (2012). Downregulation of nucleoporin 88 and 214 induced by oridonin may protect OCIM2 acute erythroleukemia cells from apoptosis through regulation of nucleocytoplasmic transport of NF‐κB. International Journal of Molecular Medicine, 30(4), 877–883. 22824908 10.3892/ijmm.2012.1067

[fsn33986-bib-0167] Yin, B. , Sheng, H. , Lin, J. , Zhou, H. , & Zhang, N. (2012). The cell death of C6 astrocytoma cells induced by oridonin and its mechanism. International Journal of Clinical and Experimental Pathology, 5(6), 562–568. 22949939 PMC3430107

[fsn33986-bib-0168] Yi‐Wen, Z. , Mei‐Hua, B. , Xiao‐Ya, L. , Yu, C. , Jing, Y. , & Hong‐Hao, Z. (2018). Effects of oridonin on hepatic cytochrome P450 expression and activities in PXR‐humanized mice. Biological and Pharmaceutical Bulletin, 41(5), 707–712. 29709908 10.1248/bpb.b17-00882

[fsn33986-bib-0169] Yong, G. U. O. , Qing‐qing, S. , & Yu‐ping, G. (2014). Anti‐leukemia effect of oridonin on T‐cell acute lymphoblastic leukemia. Journal of Sichuan University (Medical Science Edition), 45(6), 903–907. 25571712

[fsn33986-bib-0170] Yu, Y. , Fan, S. M. , Song, J. K. , Tashiro, S.‐I. , Onodera, S. , & Ikejima, T. (2012). Hydroxyl radical (· OH) played a pivotal role in oridonin‐induced apoptosis and autophagy in human epidermoid carcinoma A431 cells. Biological and Pharmaceutical Bulletin, 35(12), 2148–2159. 23207767 10.1248/bpb.b12-00405

[fsn33986-bib-0171] Zang, L. , He, H. , Xu, Q. , Yu, Y. , Zheng, N. , Liu, W. , Hayashi, T. , Tashiro, S. I. , Onodera, S. , & Ikejima, T. (2013). Reactive oxygen species H_2_O_2_ and OH, but not O_2_ − promote oridonin‐induced phagocytosis of apoptotic cells by human histocytic lymphoma U937 cells. International Immunopharmacology, 15(2), 414–423. 23352441 10.1016/j.intimp.2013.01.004

[fsn33986-bib-0172] Zang, L. , He, H. , Ye, Y. , Liu, W. , Fan, S. , Tashiro, S. I. , Onodera, S. , & Ikejima, T. (2012). Nitric oxide augments oridonin‐induced efferocytosis by human histocytic lymphoma U937 cells via autophagy and the NF‐κB‐COX‐2‐IL‐1β pathway. Free Radical Research, 46(10), 1207–1219. 22670565 10.3109/10715762.2012.700515

[fsn33986-bib-0173] Zeng, R. , Chen, Y. , Zhao, S. , & Cui, G.‐H. (2012). Autophagy counteracts apoptosis in human multiple myeloma cells exposed to oridonin *in vitro* via regulating intracellular ROS and SIRT1. Acta Pharmacologica Sinica, 33(1), 91–100. 22158107 10.1038/aps.2011.143 PMC4010261

[fsn33986-bib-0174] Zhang, C.‐L. , Wu, L.‐J. , Tashiro, S.‐I. , Onodera, S. , & Ikejima, T. (2004a). Oridonin induced A375‐S2 cell apoptosis via bax‐regulated caspase pathway activation, dependent on the cytochrome c/caspase‐9 apoptosome. Journal of Asian Natural Products Research, 6(2), 127–138. 15008459 10.1080/1028602031000147375

[fsn33986-bib-0175] Zhang, C.‐L. , Wu, L.‐J. , Tashiro, S.‐I. , Onodera, S. , & Ikejima, T. (2004b). Oridonin induces a caspase‐independent but mitochondria‐and MAPK‐dependent cell death in the murine fibrosarcoma cell line L929. Biological and Pharmaceutical Bulletin, 27(10), 1527–1531. 15467189 10.1248/bpb.27.1527

[fsn33986-bib-0176] Zhang, D. , Zhou, Q. , Huang, D. , He, L. , Zhang, H. , Hu, B. , Peng, H. , & Ren, D. (2019). ROS/JNK/c‐Jun axis is involved in oridonin‐induced caspase‐dependent apoptosis in human colorectal cancer cells. Biochemical and Biophysical Research Communications, 513(3), 594–601. 10.1016/j.bbrc.2019.04.011 30981511

[fsn33986-bib-0177] Zhang, J. , Wang, N. , Zhou, Y. , Wang, K. , Sun, Y. , Yan, H. , Han, W. , Wang, X. , Wei, B. , Ke, Y. , & Xu, X. (2021). Oridonin induces ferroptosis by inhibiting gamma‐glutamyl cycle in TE1 cells. Phytotherapy Research, 35(1), 494–503. 32869425 10.1002/ptr.6829

[fsn33986-bib-0178] Zhang, J.‐F. , Liu, J.‐J. , Liu, P.‐Q. , Lin, D.‐J. , Li, X.‐D. , & Chen, G.‐H. (2006). Oridonin inhibits cell growth by induction of apoptosis on human hepatocelluar carcinoma BEL‐7402 cells. Hepatology Research, 35(2), 104–110. 16638644 10.1016/j.hepres.2006.03.007

[fsn33986-bib-0179] Zhang, T. , Tan, Y. , Zhao, R. , & Liu, Z. (2013). DNA damage induced by oridonin involves cell cycle arrest at G2/M phase in human MCF‐7 cells. Contemporary Oncology/Współczesna Onkologia, 17(1), 38–44. 23788960 10.5114/wo.2013.33772 PMC3685353

[fsn33986-bib-0180] Zhang, W. , Shi, L. , Zhou, W. , Liu, X. , Xi, Y. , Wang, X. , Li, Y. , Xu, X. , & Tang, Y. (2023). Oridonin impedes breast cancer growth by blocking cells in S phase and inhibiting the PI3K/AKT/mTOR signaling pathway. Heliyon, 9(7), e18046. 37519735 10.1016/j.heliyon.2023.e18046 PMC10372243

[fsn33986-bib-0181] Zhang, X. , Chen, L. X. , Ouyang, L. , Cheng, Y. , & Liu, B. (2012). Plant natural compounds: Targeting pathways of autophagy as anti‐cancer therapeutic agents. Cell Proliferation, 45(5), 466–476. 22765290 10.1111/j.1365-2184.2012.00833.x PMC6496896

[fsn33986-bib-0182] Zhang, X. H. , Liu, Y. X. , Jia, M. , Han, J. S. , Zhao, M. , Ji, S. P. , & Li, A. M. (2014). Oridonin inhibits tumor growth in glioma by inducing cell cycle arrest and apoptosis. Cellular and Molecular Biology, 60(6), 29–36. 25553351

[fsn33986-bib-0183] Zhang, Y.‐H. , Wu, Y.‐L. , Tashiro, S.‐I. , Onodera, S. , & Ikejima, T. (2011). Reactive oxygen species contribute to oridonin‐induced apoptosis and autophagy in human cervical carcinoma HeLa cells. Acta Pharmacologica Sinica, 32(10), 1266–1275. 21892202 10.1038/aps.2011.92 PMC4010075

[fsn33986-bib-0184] Zhang, Y.‐W. , Bao, M.‐H. , Hu, L. , Qu, Q. , & Zhou, H.‐H. (2014). Dose‐response of oridonin on hepatic cytochromes P450 mRNA expression and activities in mice. Journal of Ethnopharmacology, 155(1), 714–720. 24933226 10.1016/j.jep.2014.06.009

[fsn33986-bib-0185] Zhang, Z. , Zhang, X. , Xue, W. , YangYang, Y. , Xu, D. , Zhao, Y. , & Lou, H. (2010). Effects of oridonin nanosuspension on cell proliferation and apoptosis of human prostatic carcinoma PC‐3 cell line. International Journal of Nanomedicine, 5, 735–742. 21042419 10.2147/IJN.S13537 PMC2962269

[fsn33986-bib-0186] Zhang, Z. Y. , Daniels, R. , & Schluesener, H. J. (2013). Oridonin ameliorates neuropathological changes and behavioural deficits in a mouse model of cerebral amyloidosis. Journal of Cellular and Molecular Medicine, 17(12), 1566–1576. 10.1111/jcmm.12124 24034629 PMC3914648

[fsn33986-bib-0187] Zhao, J. , Zhang, M. , & Chen, Y. (2012). Experimental research on the mechanisms of human multiple myeloma LP‐1 cell apoptosis induced by oridonin. Chinese Journal of Integrated Traditional and Western Medicine, 32(12), 1642–1646. 23469604

[fsn33986-bib-0188] Zhong, B. , Peng, W. , Du, S. , Chen, B. , Feng, Y. , Hu, X. , Lai, Q. , Liu, S. , Zhou, Z. W. , Fang, P. , & Wu, Y. (2022). Oridonin inhibits SARS‐CoV‐2 by targeting its 3C‐like protease. Small Science, 2(6), 2100124. 35600064 10.1002/smsc.202100124 PMC9111243

[fsn33986-bib-0189] Zhou, G. B. , Kang, H. , Wang, L. , Gao, L. , Liu, P. , Xie, J. , Zhang, F. X. , Weng, X. Q. , Shen, Z. X. , Chen, J. , & Gu, L. J. (2007). Oridonin, a diterpenoid extracted from medicinal herbs, targets AML1‐ETO fusion protein and shows potent antitumor activity with low adverse effects on t (8; 21) leukemia *in vitro* and *in vivo* . Blood, 109(8), 3441–3450. 17197433 10.1182/blood-2006-06-032250 PMC1852250

[fsn33986-bib-0190] Zhou, J. , Yun, E. J. , Chen, W. , Ding, Y. , Wu, K. , Wang, B. , Ding, C. , Hernandez, E. , Santoyo, J. , Pong, R. C. , & Chen, H. (2017). Targeting 3‐phosphoinositide‐dependent protein kinase 1 associated with drug‐resistant renal cell carcinoma using new oridonin analogs. Cell Death & Disease, 8(3), e2701. 28333136 10.1038/cddis.2017.121 PMC5386527

[fsn33986-bib-0191] Zhu, H. Q. , Zhang, C. , Guo, Z.‐Y. , Yang, J.‐M. , Guo, J.‐H. , Chen, C. , Yao, Q.‐H , Liu, F. , Zhang, Q.‐W. , & Gao, F.‐H. (2019). Oridonin induces Mdm2‐p60 to promote p53‐mediated apoptosis and cell cycle arrest in neuroblastoma. Cancer Medicine, 8(11), 5313–5326. 31339234 10.1002/cam4.2393 PMC6718599

[fsn33986-bib-0192] Zhu, M. I. N. , Hong, D. U. N. , Bao, Y. , Wang, C. , & Pan, W. (2013). Oridonin induces the apoptosis of metastatic hepatocellular carcinoma cells via a mitochondrial pathway. Oncology Letters, 6(5), 1502–1506. 24179549 10.3892/ol.2013.1541 PMC3813803

[fsn33986-bib-0193] Zhu, Y. , Lehao, W. , Zhao, Y. , Wang, Z. , Jihong, L. , Yang, Y. , Xiao, H. , & Zhang, Y. (2022). Discovery of oridonin as a novel agonist for BRS‐3. Phytomedicine, 100, 154085. 35405616 10.1016/j.phymed.2022.154085

